# Functionalized [2.2]Paracyclophanedienes as Monomers for Poly(*p*-phenylenevinylene)s

**DOI:** 10.1021/acsmacrolett.3c00714

**Published:** 2024-01-08

**Authors:** Arielle Mann, Chengyuan Wang, Bianca L. Dumlao, Marcus Weck

**Affiliations:** Department of Chemistry and Molecular Design Institute, New York University, New York, New York 10003, United States

## Abstract

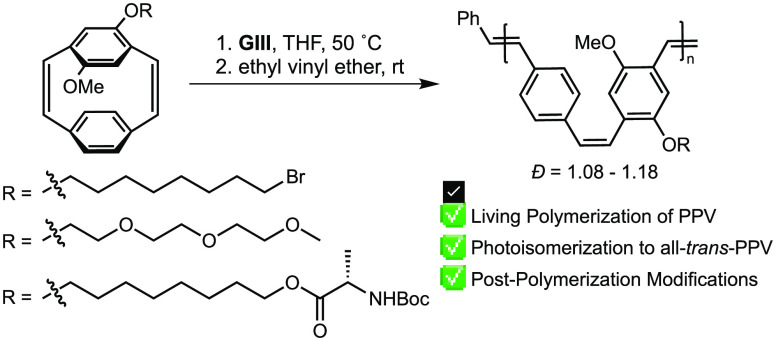

Poly(*p*-phenylenevinylene)s (PPVs) featuring complex side-chains, to date, have only been synthesized by nonliving polymerization methods which have no control over PPV molecular weights, dispersities, or end groups. [2.2]Paracyclophane-1,9-diene (pCpd) has gained attention as a monomer for its ability to be ring-opened to PPV in a living fashion. pCpd, an organic cyclic scaffold with planar chirality, has seen minimal structural diversity due to the harsh reaction conditions required to afford the highly strained compound. Herein, we introduce a general method to overcome this by targeting the synthesis of a monohydroxy-pCpd via mono-demethylation of a dialkoxy-pCpd. The monohydroxy-pCpd can then be functionalized easily, which we demonstrate using three distinct side-chains with four moieties commonly incorporated in conjugated polymers: an alkyl bromide, an oligo(ethylene glycol) chain, an enantiomerically pure side-chain, and a Boc-protected amine. These monofunctionalized-pCpds were investigated as monomers in the ring-opening metathesis polymerization (ROMP) to afford functionalized PPVs in a living manner. The functional-group-containing PPVs are synthesized with full control over their end groups, repeat units, and dispersities. The feasibility of post-polymerization modifications to incorporate any desired moiety to PPV fabricated by this method was demonstrated using an azide–alkyne click reaction. All synthesized PPVs were soluble in organic solvents and display the same fluorescent emission, indicating their conjugated backbones are unaltered.

Organic conducting polymers have seen remarkable progress and applications since the initial discovery of doped poly(acetylene).^1−3^ Poly(*p*-phenylenevinylene) (PPV), a π-conjugated polymer, has been extensively explored being the first active layer in an organic light-emitting diode (OLED).^4^ PPVs find applications in OLEDS,^5,6^ transistors, and organic photovoltaic cells (OPVS)^7^ and are used for their fluorescent properties in areas such as bioimaging and drug delivery.^8−12^ An attractive feature of PPV is that alkyl or alkoxy side-chains are easily appended at the 2,5-positions allowing for solution processing of the polymer.^1,13^ When *para*-alkoxy side-chains are incorporated, they increase the electron density of the conjugated backbone causing a favorable bathochromic shift of the PPV’s optical properties.^14,15^ Additional moieties at the terminus of the side-chains, such as polar groups, salts, or biologically relevant moieties, further engineer the polymer’s solubilizing media and applications.^16−22^

Common methods for the synthesis of PPVs have been well reviewed and include precursor routes, step-growth polymerizations, and polycondensations. These methods, however, have no control over molecular weights and tend to incorporate backbone defects. Nevertheless, they are currently the only way to incorporate functional side-chains into PPV.^13,23,24^ Polymer chemists have strived to improve the synthetic strategies of PPVs by turning to olefin metathesis polymerizations: acyclic diene metathesis (ADMET) or the living ring-opening metathesis polymerization (ROMP).^23,25,26^ ROMP, in particular, introduces a living chain-growth polymerization allowing for regioregular and defect free polymers with control over the polymer molecular weights, end groups, and dispersities which has been shown to be critical to tune and control optical properties.^25,27,28^ [2.2]Paracyclophane-1,9-diene (pCpd), **5**, a highly strained layered organic scaffold with benzene “decks” and vinylene “bridges” has gained significant attention since being investigated as a monomer for the living ROMP to yield PPV (Scheme 1).^25,29−33^

**Scheme 1:**
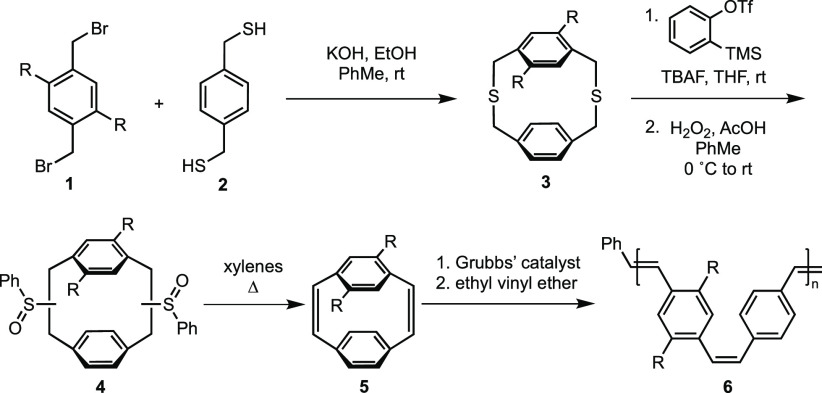
Synthesis and ROMP of [2.2]Paracyclophane-1,9-diene^30^ (R = H, Alkyl, or Alkoxy)

The pCpd scaffold has been synthesized to incorporate alkyl^34^ and alkoxy^35,36^ side-chains, benzothiadiazoles,^37−39^ and a bromine atom for a palladium-catalyzed cross-coupling reaction.^40,41^ These limited structural modifications to pCpd’s decks, especially when compared to its structural analogue [2.2]paracyclophane,^42,43^ can be attributed to the harsh reaction conditions required to install the alkene “bridges” (Scheme 1) and the sensitivity of the carbon–carbon double bonds to reaction conditions.^30^ As a result, this dearth of structural modifications to pCpd limits the applications of PPVs synthesized from these monomers. This contribution presents a general strategy toward functionalized PPVs by introducing a monohydroxy pCpd. Through the optimization of deprotection conditions of 4,7-dimethoxy-pCpd, **7**, the hydroxy-containing pCpd can be functionalized by a variety of side-chains. These functionalized pCpds can be polymerized in a living fashion via ROMP, allowing for control of PPVs molecular weights, end groups, optical properties, and post-polymerization modifications.

As alkoxy substituted pCpds with various substitution patterns are easily accessible,^35,36^ a methodology to use these compounds would be advantageous. Therefore, we decided to start our methodology with dimethoxy-pCpd **7** (Scheme 2). Adding 3 equiv of boron tribromide to **7** at −78 °C and stirring the reaction mixture at room temperature for 2.5 h afforded OH/OMe-pCpd **8** in good yields with some recovery of starting material. To ensure the alkene bridges tolerated the acidic conditions of the deprotection, **8** was treated with iodomethane under basic conditions to reinstall the methyl ether. The matching spectroscopic data collected for the product compared to dimethoxy-pCpd **7** validate our strategy for the functional handle-containing hydroxy-pCpd. Attempts to make a hydroquinone-pCpd only resulted in the overoxidized quinone-pCpd **9**, which has previously been reported.^44^ All pCpds in this investigation were used as racemic mixtures of the planar chiral enantiomers *R*_p_ and *S*_p_ (Supporting Information section 3). For clarity, only the *S*_p_ pCpd is depicted in all figures.

**Scheme 2:**
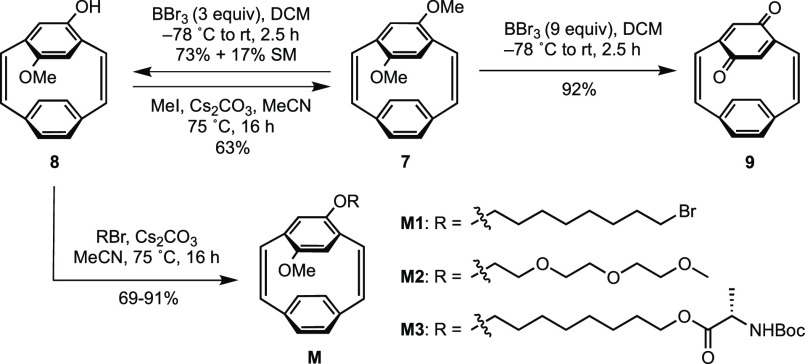
Treatment of 4,7-Dimethoxy-[2.2]paracyclophane-1,9-diene with Boron Tribromide and Its Subsequent Functionalization

We envision that a hydroxy-containing pCpd scaffold can be used for various applications such as ligands for chiral catalysts,^45^ chiroptics,^46^ pharmaceuticals,^47^ or materials science.^45^ We focused our investigation on the application of **8** as a monomer for the controlled polymerization of PPVs by designing three pCpd monomers featuring side-chains commonly found in conjugated polymers. Monohydroxy-pCpd **8** was treated with 1,8-dibromooctane, diethylene glycol 2-bromoethyl methyl ether, or bromooctyl-l-Ala-Boc using cesium carbonate as a base in acetonitrile to form OctBr-pCpd **M1**, tri(ethylene glycol) TEG-pCpd **M2**, and OctAlaBoc-pCpd **M3**, respectively, in good yields (Scheme 2). Both **M1** and **M3** are functional monomers for post-polymerization modifications using the bromide or the deprotected amine.^9,20,48−50^**M3** also demonstrates the ease by which enantiomerically pure side-chains can be incorporated which affects the optical properties of PPV^51^ and is economically beneficial as a late-stage modification. **M2**, instead, contains an oligo(ethylene glycol) moiety that is commonly used in conjugated polymers for its hydrophilicity, high polarity, and ion-conductivity allowing for a multitude of applications.^16,50,52^

The three functionalized pCpd monomers were then investigated for their reactivity in ROMP using Grubbs’ third generation initiator (**GIII**) in THF at 50 °C. We observed a linear relationship for each of the monomer to initiator ratios ([**M**]/[**GIII**]) plotted with the molecular weights (*M*_n_) (Figure 1). The *M*_n_ was collected from gel-permeation chromatography (GPC) in THF, but a more accurate value for these rigid rod polymers is obtained by ^1^H NMR spectroscopy from end-group analysis through the integration of the terminal vinyl group and the methylene groups attached to the oxygen atom on PPV’s backbone. During ROMP, full consumption of each monomer was confirmed by monitoring the polymerizations *in situ* by ^1^H NMR spectroscopy (Supporting Information section 4.1). All polymers have low dispersities (*Đ* = 1.08–1.18) demonstrating the impact of the living ROMP on polymer properties (Table 1). The 10mer of each polymer (**P1a**, **P2a**, and **P3a**) was further characterized by matrix-assisted laser desorption/ionization time-of-flight mass spectrometry (MALDI-TOF-MS). For each polymer, a series of major peaks separated by the mass of the monomer are observed. Additionally, the masses are consistent with PPVs capped with a phenyl group from the **GIII** initiator on one end and a vinyl group from the termination with ethyl vinyl ether at the other terminus (Supporting Information section 4.6). Diblock copolymers were also synthesized using dioctyloxy-pCpd, and consistent shifts in molecular weights were observed (Supporting Information section 4.4). All of these results are consistent with a living polymerization for each functionalized pCpd monomer.

**Figure 1:**
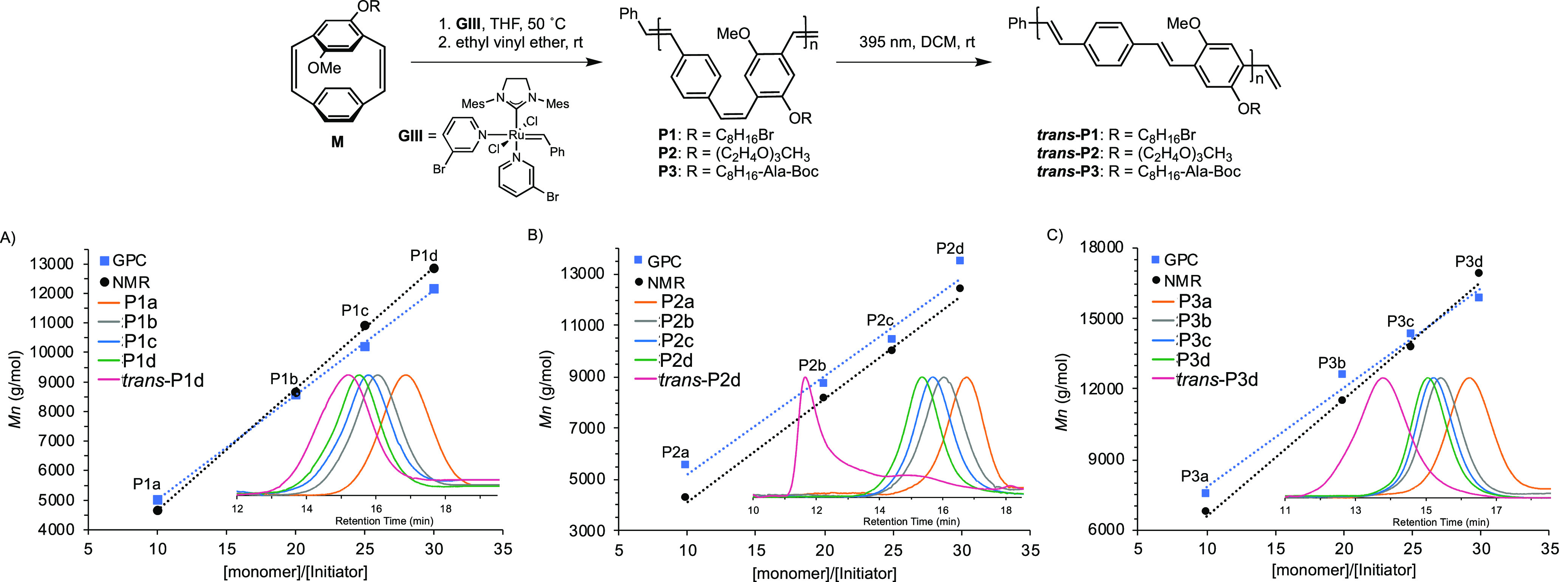
GPC and NMR-spectroscopy-obtained molecular weights as a function of [monomer]/[**GIII**] of (A) OctBr-pCpd **M1**, (B) TEG-pCpd **M2**, and (C) OctAlaBoc-pCpd **M3**. The GPC traces collected in THF of the polymers using the RI detector are also shown (inset).

**Table 1:** GPC Data and Optical Characterization of All of the New PPVs

PPV	[**M**]/[**GIII**]	*M*_n,calc_^a^	*M*_n,GPC_^b^	*M*_n,NMR_^c^	*Đ*	% yield	abs λ_max_ (nm)^d^	em. λ_max_ (nm)^d^
**P1a**	10	4518	5000	4700	1.17	89	417	524
**P1b**	20	8932	8600	8700	1.18	89	456	524
**P1c**	25	11139	10200	1100	1.16	94	458	524
**P1d**	30	13346	12200	12800	1.18	92	459	524
***trans*-P1d**	-	13346	15800	12600	1.26	97	467	524
**P2a**	10	4069	5500	4200	1.08	66	415	521
**P2b**	20	8034	8700	8100	1.16	88	421	521
**P2c**	25	10016	10400	10000	1.18	85	441	521
**P2d**	30	11998	13400	12300	1.18	93	456	521
***trans*-P2d**	-	11998	250900	12300	1.28	87	467	521
**P3a**	10	5601	7500	6800	1.12	76	421	522
**P3b**	20	11098	12600	11500	1.11	78	423	522
**P3c**	25	13847	14300	13700	1.15	78	425	522
**P3d**	30	16595	15900	16700	1.12	69	426	522
***trans*-P3d**	-	16595	40800	17300	1.42	93	467	522

a Calculated based on the targeted degree of polymerization noted in [**M**]/[**GIII**].

b *M*_n_ GPC values were determined against polystyrene standards.

c *M*_n_ NMR values estimated through relative integration of the terminal vinyl resonance and the side-chain protons.

d Measurements were done in dilute solutions of chloroform.

The ROMP of pCpd with **GIII** yields a *cis*,*trans*-PPV (**P1**–**3**) as only one of the vinylene bridges are opened during the polymerization resulting in a *trans*-vinylene linkage while the untouched *cis*-vinylene of the monomer remains.^53^ Due to overlapping signals in the NMR spectra, the exact *cis*-to-*trans* ratio cannot be determined at this time. The *cis*,*trans*-PPV, however, can be photoisomerized with UV light to form the all-*trans*-PPV, which is critical as all-*trans*-PPVs perform better in devices due to the longer conjugation length.^15^ PhotoNMR spectroscopy experiments (λ = 395 nm) were performed to monitor the post-polymerization isomerization in DCM-*d*_2_ of a 30mer for each polymer synthesized: **P1d**, **P2d**, and **P3d**. In each case, the isomerization was complete within 2 h and the peaks of the conjugated backbone (δ 7.7–6.4 ppm) converge upfield, consistent with signals associated with *trans*-vinylene protons.^29,35^ Additionally, the methylene groups attached to the oxygen atom on PPV’s backbone significantly decreased for **P1d** and **P2d** and disappeared for **P3d** around δ 3.5 ppm, while the *trans* stereoisomer peak around δ 4.2 ppm became more prominent (Supporting Information section 4.3). GPC analyses of *trans*-**P1d**, *trans*-**P2d**, and *trans*-**P3d** showed a lower retention time in comparison to the *cis*,*trans*-polymer, which is consistent with the expected coil-to-rod transition (Figure 1). In the case of the TEG-containing PPV, *trans*-**P2d** is seen to aggregate (Figure 1B, inset), which we suggest is due to the highly polar ethylene glycol side-chain encapsulating the significantly less soluble, now more rodlike PPV backbone. This observation demonstrates potential applications for the triggerable assembly of materials using this methodology.^54^

The optical properties of the PPVs were measured in dilute solutions of chloroform. All PPVs showed broad absorption bands in solution ranging from 417 to 467 nm (Figure 2). Bathochromic shifts of the absorbance maximum are a result of the conjugation length increasing either by increasing the number of repeat units or by photoisomerizing the *cis*,*trans*-PPV to the all-*trans*-PPV. Additionally, the photoluminescence spectra show nearly identical emissions around 522 nm for all polymers, regardless of side-chain, and match the characterization data of similar PPVs synthesized by dialkoxy-pCpds.^55^

**Figure 2:**
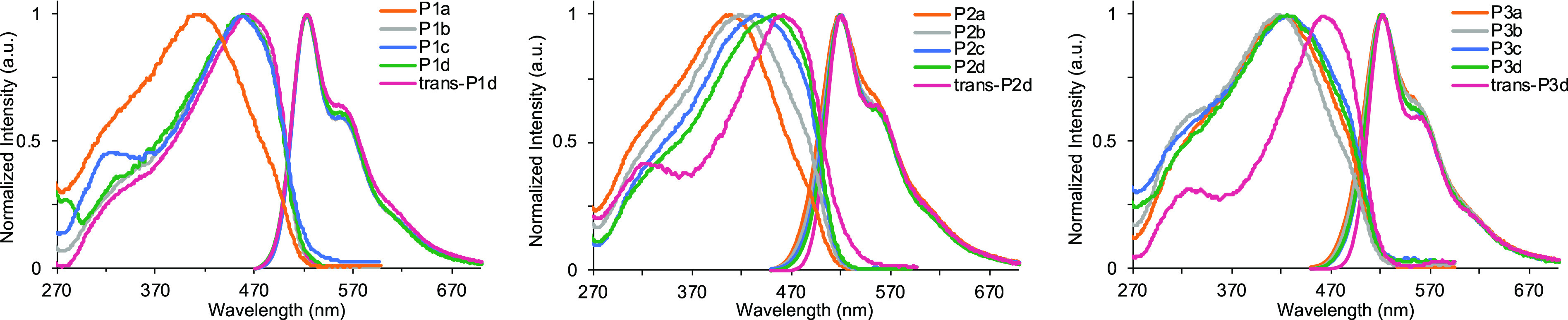
Absorbance and emission spectra of PPV polymers **P1a**–**d** and *trans*-**P1d** (left), **P2a**–**d** and *trans*-**P2d** (center), and **P3**a****–****d**** and *trans*-**P3d** (right).

Finally, using our functional PPVs, post-polymerization modifications were explored as a general method to attach any desired side-chain to low dispersed PPVs. Specifically, we chose the alkyne–azide click reaction which is a commonly employed strategy that has already been reported for PPVs synthesized by nonliving methods.^49,56^ Brominated polymer **P1a** was treated with sodium azide, affording azide-functionalized **P1a-azide**. The azide polymer was then treated with propargyl coumarin **10** using the copper(I)-catalyzed alkyne–azide cycloaddition (CuAAC) (Scheme 3). A coumarin moiety was chosen as the label because it is easily analyzed and has found extensive use in cell recognition, sensing, and labeling.^57−59^ The chemical manipulation was confirmed by ^1^H NMR and FT-IR spectroscopies, GPC, and MALDI-TOF-MS. Additionally, the optical properties of **P1-azide** and **P1-click** are nearly identical to those of **P1a**, indicating that the conjugated backbone remains intact.

**Scheme 3:**
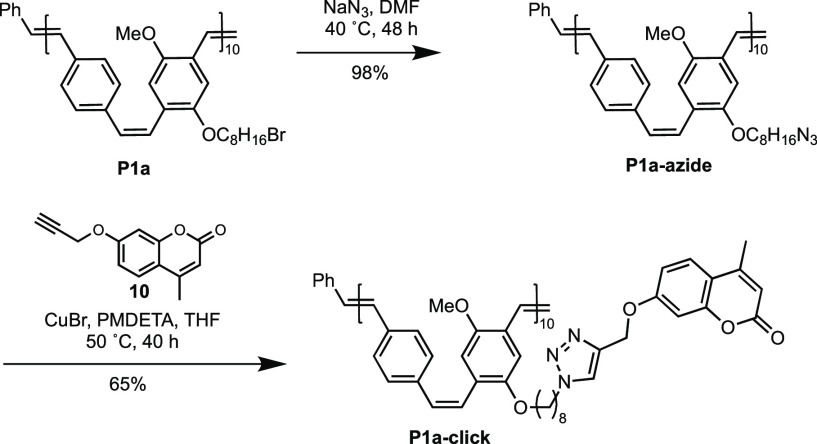
Post-Polymerization Modification and Click Reaction of **P1a** with 4-Methyl-7-(propargyloxy)coumarin **10**

In summary, we have developed a methodology for the synthesis of a hydroxy-containing pCpd and its substitution with functional side-chains as a general method toward side-chain-functionalized PPVs. The late-stage monomer functionalization allows for the inclusion of functional group containing side-chains that otherwise do not survive the synthesis of the pCpd. Additionally, our strategy is economically beneficial for the incorporation of expensive enantiomerically pure alkanes or bioorthogonal containing side-chains. All pCpds were found to polymerize via ROMP in a controlled fashion, affording polymers with low dispersities and full control over repeat units, molecular weights, end groups, and consistent optical properties. Further, the ease of post-polymerization modifications using PPVs fabricated by this strategy was demonstrated using an alkyne–azide click reaction. Finally, as demonstrated with the oligo(ethylene glycol)-PPV **P2**, these polymers can also be used for controlled triggerable assemblies by photoisomerization of the backbones’ alkenes. We view our strategy as a key advance for conducting polymers with potential applications in advanced optical materials.

## Supporting Information Available

Full experimental details, fabrication of diblock copolymers, NMR spectra, optical spectra, and chiral HPLC spectra (PDF)
