# Supplementary material for: Functionalized [2.2]Paracyclophanedienes as Monomers for Poly(p-phenylenevinylene)s
Source: ACS Macro Lett. 2024 Jan 8;13(2):112–7. doi: 10.1021/acsmacrolett.3c00714 (PMC10883051; doi:10.1021/acsmacrolett.3c00714)

Supporting Information

Functionalized [2.2]Paracyclophanedienes as Monomers for Poly(*p*-Phenylenevinylene)s

Arielle Mann, Chengyuan Wang, Bianca L. Dumlao, Marcus Weck*

Molecular Design Institute and Department of Chemistry, New York University
New York, NY 10003, USA

*To whom correspondence should be addressed: marcus.weck@nyu.edu

Table Of Contents

1. Materials and Methods S2
2. Small Molecule Synthesis S3
3. Chiral HPLC S7
4. Polymerizations S10
4.1 In-situ ^{1}H NMR experiments S10
4.3 Photoisomerization S14
4.4 Polymerization of Diblocks S18
4.5 Post-polymerization modification S21
4.6 MALDI-TOF-MS S25
5. References S27
6. NMR Spectra S28

1. Materials and Methods

All chemicals were purchased from Oakwood Chemicals, TCI Chemicals, or Millipore Sigma and used as received unless otherwise indicated. All reactions were carried out under ambient conditions unless otherwise noted. Flash column chromatography was performed using silica gel 60 Å (230-400 mesh) from Sorbent Technologies.

NMR spectroscopy characterizations were conducted at 25 ˚C on a Bruker Avance 400 MHz, 500 MHz, 600 MHz, or 800 MHz spectrometers. Chemical shifts are reported in ppm and referenced to solvent residual peaks. Splitting patterns are reported as broad signal (br), singlet (s), doublet (d), doublet of doublets (dd), triplet (t), quartet (q) and multiplet (m).

Mass spectra of samples in methanol were acquired with an Agilent 6224 Accurate-Mass TOF/LC/MS Spectrometer using an ESI ion-source.

For Chiral HPLC, the samples were separated and analyzed by an Agilent 1260 Infinity HPLC equipped with CHIRALPAK IA-3 column or OD-H01 column.

Gel-permeation chromatography (GPC) characterizations were obtained from a Shimadzu pump coupled to a Shimadzu UV and RI detectors with tetrahydrofuran (THF) (stabilized with BHT) as the mobile phase. The injection volume was 50 µL and the flow rate was 1 mL/min on a Shimadzu column set (100, 1000, 100,000 Å, linear mixed bed). The GPC instruments were calibrated using poly(styrene) standards (EasiCal, Agilent Technologies, Santa Clara, CA) and characterizations were carried out at 25 °C. M_n and $Đ$ represent number average molecular weight and dispersity, respectively.

PhotoNMR was conducted using NewEra PhotoNMR tube NE-379-5-Br which has an inner cell insert for an optical cable. A coaxial cable (NMR-Fiber), connected to a high-power LED illuminator at 395 nm was inserted and the sample was manually inserted into a Bruker NMR (400 MHz) at 25 ˚C.

Fourier Transform Infrared (FT-IR) Spectroscopy were recorded on a ThermoFisher Scientific Nicolete 6700 FT-IR spectrometer. Samples were measured as a thin film formed from a solution after solvent evaporation.

Matrix-Assisted Laser Desorption Ionization Time-Of-Flight (MALDI-TOF) mass spectrometry was recorded on a Bruker MALDI-TOF UltraFleXtreme spectrometer using dithranol (10 mg/mL) as the matrix.

Absorption spectra were obtained using a Cary 100 UV-VIS Spectrophotometer by Agilent Technologies. Fluorescence spectra were collected on a QuantaMaster 40 Photon Technology International spectrofluorometer equipped with Xenon lamp source, emission and excitation monochromators, excitation correction unit, and PMT detector. All measurements were conducted at 25.0 ± 0.1 °C maintained by a Quantum Northwest cuvette temperature controller. Emission and excitation spectra were corrected for the wavelength-dependent response and wavelength dependent lamp intensity.

2. Small Molecule Synthesis

4,7-Dimethoxy-[2.2]Paracyclophane-1,9-diene (**7**),[1] 4,7-dioctyloxy-[2.2]Paracyclophane-1,9-diene[2] (**M4**), and 4-methyl-7-(propargyloxy)coumarin (**10**)[3] were prepared by reported procedures.

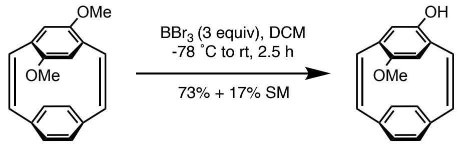

OH/OMe-pCpd **8**:
To an oven dried 100 mL Schlenk flask evacuated and filled with argon three times, dimethoxy-pCpd (**7**) (0.496 g, 1.87 mmol, 1 equiv) was dissolved in 75 mL of dry DCM and cooled in an acetone and dry ice bath. Using a syringe pump, a 1M BBr_3 solution in DCM (5.63 mL, 3 equiv) was added over 20 mins and the reaction mixture turned dark red. The reaction was warmed to room temperature and allowed to stir for an additional 2.5 hours before it was placed in an ice water bath and DI water (5 mL) was added slowly. The mixture was filtered through a plug of celite and rinsed with water and DCM. The organic layer was then extracted, dried over $MgSO_4$, filtered, and concentrated under reduced pressure. The crude product was adhered to silica gel and purified using flash column chromatography on silica gel using a gradient eluent of 3:1 to 1:1 hexanes/DCM to afford **8** as an off white solid (0.342 g, 1.37 mmol, 73%) as well as 84 mg of starting material (17%) was recovered.
^{1}H NMR (400 MHz, $CDCl_3$) δ 7.33 (d, *J*= 9.9, 1H), 7.16 (d, *J*= 10.1, 1H), 7.02 (dd, *J*= 7.9, 0.9, 1H), 6.90 (d, *J*= 10.2, 1H), 6.85 (d, *J*= 9.9, 1H), 6.8 (d, *J*= 7.9, 1H), 6.58 (d, *J*= 7.9, 1H), 6.44 (d, *J*= 7.4, 1H), 5.76 (s, 1H), 5.71 (s, 1H), 4.26 (br s, 1H), 3.69 (s, 3H).
^{13}C NMR (125 MHz, $CDCl_3$) δ 152.9, 148.2, 140.0, 138.6, 137.5, 136.1, 134.2, 132.3, 131.5, 131.4, 128.0, 127.9, 126.4, 124.5, 124.3, 117.5, 56.1.
HRMS (ESI) *m/z* calculated for $C_{17}H_{14}O_2Na$ $(M+Na)^+$ 273.0886, found 273.0909.

Note: The monodemethylated product **2** and the quinone-pCpd **3** are challenging to separate by column chromatography as they easily coelude (Figure S1). Monitoring the reaction by TLC, the reaction to afford **2** is stopped while starting material is still seen to reduce the amount of **3**, making the purification simpler.

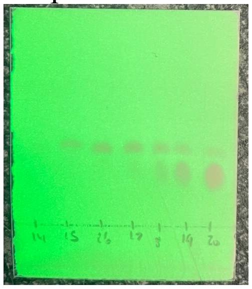

Figure S1. TLC of OH/OMe-pCpd **2** (bottom spot) and quinone-pCpd **3** (top spot) run in DCM.

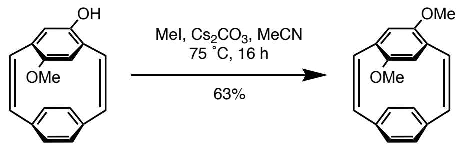

8 **7**

OMe-pCpd **7**:

To an oven dried 100 mL Schlenk flask with an argon atmosphere, OH/OMe-pCpd **8** (0.050 g, 0.20 mmol, 1 equiv) and cesium carbonate (0.195 g, 0.50 mmol, 2.5 equiv) were added, dissolved in anhydrous acetonitrile (5 mL), and allowed to stir at room temperature for 10 mins. Iodomethane (0.03 mL, 2 equiv) was then added the reaction was heated to 75 ˚C for 16 hours. The reaction was cooled to room temperature and the solvent was removed under reduced pressure. The crude reaction was dissolved in DCM (40 mL), washed with water (3 x 30 mL) and brine, dried over $MgSO_4$, filtered, and adhered to silica for flash column chromatography on silica gel using an eluent of 2:1 hexanes/DCM. The product was afforded as a white oil (0.053 g, 63%). The spectroscopic data were consistent with the previously reported data for the pCpd.[1]

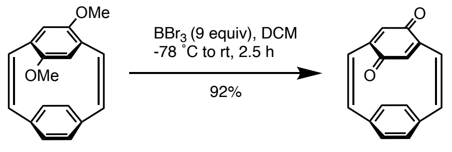

7 **9**

Quinone-pCpd **9**:

To an oven dried 100 mL Schlenk flask evacuated and filled with argon three times, dimethoxy-pCpd (**7**) (0.200 g, 0.76 mmol, 1 equiv) was dissolved in 30 mL of dry DCM and cooled in an acetone and dry ice bath. Using a syringe pump, a 1M BBr_3 solution in DCM (6.81 mL, 9 equiv) was added over 20 mins and the reaction mixture turned dark red. The reaction was warmed to room temperature and allowed to stir for an additional 2.5 hours before it was placed in an ice water bath and DI water (5 mL) was added slowly. The mixture was filtered through a plug of celite and rinsed with water and DCM. The organic layer was then extracted, dried over $MgSO_4$, filtered, and concentrated under reduced pressure. The crude product was adhered to silica gel and purified using flash column chromatography on silica gel using DCM to afford the product as a yellow solid (0.163 g, 0.70 mmol, 92%).

^{1}H NMR (400 MHz, $CDCl_3$) δ 7.28 (d, *J*= 10.3, 2H), 6.89 (s, 4H), 6.64 (dd, *J*= 10.3, 1.8, 2H), 5.82 (d, *J*= 1.9, 2H).

^{13}C NMR (125 MHz, $CDCl_3$) δ 187.8, 147.6, 138.4, 137.5, 134.8, 132.8, 130.9, 130.2.

HRMS (ESI) *m/z* calculated for $C_{16}H_{10}O_2Na$ $(M+Na)^+$ 257.0573, found 257.0536.

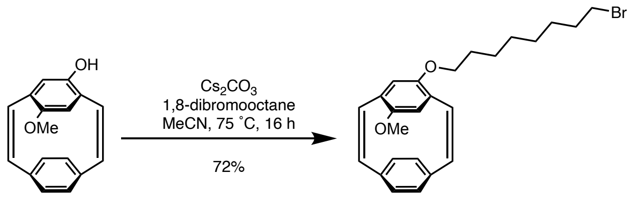

8 **M1**

$C_8H_{16}Br$/OMe-pCpd **M1**:

To an oven dried 100 mL Schlenk flask with an argon atmosphere, OH/OMe-pCpd **8** (0.403 g, 1.60 mmol, 1 equiv) and cesium carbonate (1.302 g, 4.00 mmol, 2.5 equiv) were added, dissolved

in anhydrous acetonitrile (80 mL), and allowed to stir at room temperature for 10 mins. 1,8-dibromooctane (0.88 mL, 4.80 mmol, 3 equiv) was then added dropwise over 5 mins and the reaction was heated to 75 ˚C for 16 hours. The reaction was cooled to room temperature and the solvent was removed under reduced pressure. The crude reaction was dissolved in DCM (40 mL), washed with water (3 x 30 mL) and brine, dried over $MgSO_4$, filtered, and adhered to silica for flash column chromatography on silica gel using a gradient eluent of 1:0 to 1:1 hexanes/DCM. The product was afforded as a clear oil (0.513 g, 72%).
^{1}H NMR (400 MHz, $CDCl_3$) δ 7.14 (t, *J*= 10.2, 2H), 6.91 (d, *J*= 10.3, 2H), 6.81 (m, 2H), 6.48 (d, *J*= 7.8, 2H), 5.79 (s, 1H), 5.77 (s, 1H), 3.78 (m, 2H), 3.67 (s, 3H), 3.41 (t, *J*= 6.8, 2H), 1.87 (quint, *J*= 7.1, 2H), 1.72 (quint, *J*= 6.81, 2H), 1.45 (m, 4H), 1.36 (m, 4H).
^{13}C NMR (125 MHz, $CDCl_3$) δ 152.8, 152.5, 138.36, 138.31, 136.0, 135.7, 134.5, 134.2, 131.5, 131.3, 127.7, 127.5, 127.4, 126.9, 120.6, 119.0, 69.2, 56.0, 34.1, 32.9, 29.6, 29.3, 28.8, 28.2, 26.3.
HRMS (ESI) *m/z* calculated for $C_{25}H_{29}O_2BrNa$ $(M+Na)^+$ 463.1243, found 463.1311.

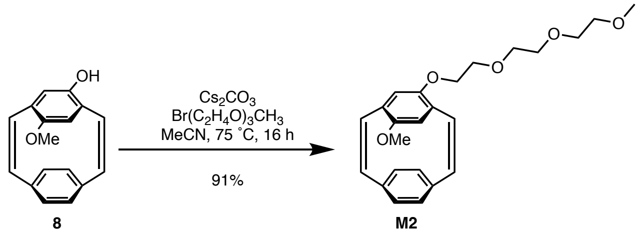

TEG/OMe-pCpd **M2**:
To an oven dried 100 mL Schlenk flask with an argon atmosphere, OH/OMe-pCpd **8** (0.400 g, 1.60 mmol, 1 equiv) and cesium carbonate (1.302 g, 4.00 mmol, 2.5 equiv) were added, dissolved in anhydrous acetonitrile (30 mL), and allowed to stir at room temperature for 10 mins. Diethylene glycol 2-bromoethyl methyl ether (0.363 g, 1.60 mmol, 1 equiv) was then added and the reaction was heated to 75 ˚C for 16 hours. The reaction was cooled to room temperature and the solvent was removed under reduced pressure. The crude reaction was dissolved in DCM (40 mL), washed with water (3 x 30 mL) and brine, dried over $MgSO_4$, filtered, and adhered to silica for flash column chromatography on silica gel using an eluent of 4:1 hexanes/ethyl acetate. The product was afforded as a brown oil (0.575 g, 91%).
^{1}H NMR (400 MHz, $CDCl_3$) δ 7.13 (t, *J*= 11.0, 2H), 6.90 (d, *J*= 10.2, 2H), 6.83 (dd, *J*= 19.7, 7.8, 2H), 6.47 (m, 2H), 5.82 (s, 1H), 5.77 (s, 1H), 3.96 (m, 2H), 3.82-3.73 (m, 4H), 3.73-3.64 (m, 4H), 3.67 (s, 3H), 3.55 (m, 2H), 3.38 (s, 3H).
^{13}C NMR (125 MHz, $CDCl_3$) δ 153.0, 152.2, 138.3, 136.1, 135.9, 134.4 134.1, 131.6, 131.2, 127.8, 127.7, 127.3, 126.9, 120.8, 118.9, 72.1, 71.0, 70.8, 70.7, 70.0, 69.0, 59.1, 55.9.
HRMS (ESI) *m/z* calculated for $C_{24}H_{28}O_5Na$ $(M+Na)^+$ 419.1829, found 419.1893.

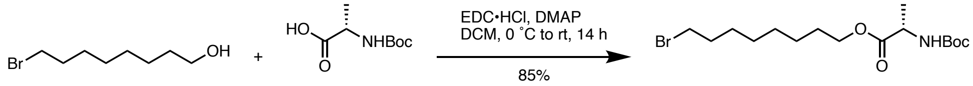

Boc-L-Alanine Br-Oct-Ala-Boc

Br-Oct-AlaBoc:
To an oven dried 500 mL round bottom flask, boc-L-alanine (5.00 g, 26.43 mmol) and DMAP (0.293 g, 2.40 mmol) were combined. The solids were evacuated three times and filled with Argon and then dissolved in 75 mL of dichloromethane. To the solution, 8-bromooctane-1-ol (4.12 mL, 24.00 mmol) was then added. The solution was cooled to 0 ºC and EDC•HCl (5.07 g, 26.40 mmol)

was added scoop wise. The mixture was left stirring at room temperature for 14 hours after which it was quenched with saturated $NH_4Cl_{(aq)}$ and extracted with ethyl acetate. The organic layer was concentrated and purified by flash column chromatography on silica using 5:1 hexanes/ethyl acetate as the eluent gave a clear, yellow oil (8.53 g, 85% yield).
^{1}H NMR (400 MHz, $CDCl_3$) δ 5.03 (s, 1H), 4.30 (t, *J*= 6.8, 1H), 4.13 (m, *J*= 4.9, 2H), 3.40 (t, *J*= 6.8, 2H), 1.85 (q, *J*= 7.2, 2H), 1.64 (t, *J*= 6.9, 2H), 1.44 (s, 12H), 1.43-1.30 (br, 8H).
^{13}C NMR (125 MHz, $CDCl_3$) δ 173.6, 155.2, 79.9, 65.4, 49.3, 34.0, 32.8, 29.1, 28.7, 28.6, 28.4, 28.1, 25.8, 18.9.
HRMS (ESI) *m/z* calculated for $C_{16}H_{30}BrNO_4Na$ $(M+Na)^+$ 402.125, found 402.1243.

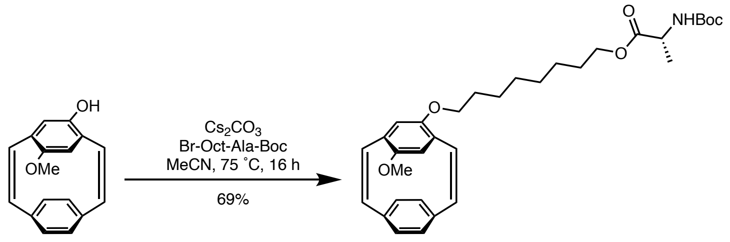

8
M3

OctAlaBoc/OMe-pCpd **M3**:
To an oven dried 100 mL Schlenk flask with an argon atmosphere, OH/OMe-pCpd **8** (0.400 g, 1.60 mmol, 1 equiv) and cesium carbonate (1.302 g, 4.00 mmol, 2.5 equiv) were added, dissolved in anhydrous acetonitrile (30 mL), and allowed to stir at room temperature for 10 mins. Br-oct-ala-boc (0.608 g, 1.60 mmol, 1 equiv) was then added and the reaction was heated to 75 °C for 16 hours. The reaction was cooled to room temperature and the solvent was removed under reduced pressure. The crude reaction was dissolved in DCM (40 mL), washed with water (3 x 30 mL) and brine, dried over $MgSO_4$, filtered, and adhered to silica for flash column chromatography on silica gel using an eluent of 5:1 hexanes/ethyl acetate. The product was afforded as a brown oil (0.606 g, 69%).
^{1}H NMR (400 MHz, $CDCl_3$) δ 7.13 (t, 2H), 6.90 (d, *J*= 10.2, 2H), 6.80 (m, 2H), 6.47 (d, *J*= 7.9, 2H), 5.79 (s, 1H), 5.77 (s, 1H), 5.03 (br s, 1H), 4.30 (br t, *J*= 7.3, 1H), 4.13 (m, 2H), 3.78 (m, 2H), 3.67 (s, 3H), 1.76-1.60 (m, 4H), 1.44 (s, 12H), 1.40-1.32 (br, 8H).
^{13}C NMR (125 MHz, $CDCl_3$) δ 173.6, 155.3, 152.7, 152.5, 138.3, 138.3, 136.0, 135.7, 134.5, 134.2, 131.5, 131.3, 127.7, 127.5, 127.4, 126.9, 120.6, 119.0, 79.9, 69.2, 65.5, 56.0, 49.4, 29.7, 29.4, 29.3, 28.6, 28.4, 26.2, 25.9, 18.9,
HRMS (ESI) *m/z* calculated for $C_{33}H_{43}NO_6Na$ $(M+Na)^+$ 572.2983, found 572.3063.

3. Chiral HPLC

General procedure: Compounds were individually dissolved in a 1:1 mixture of hexanes and isopropanol.

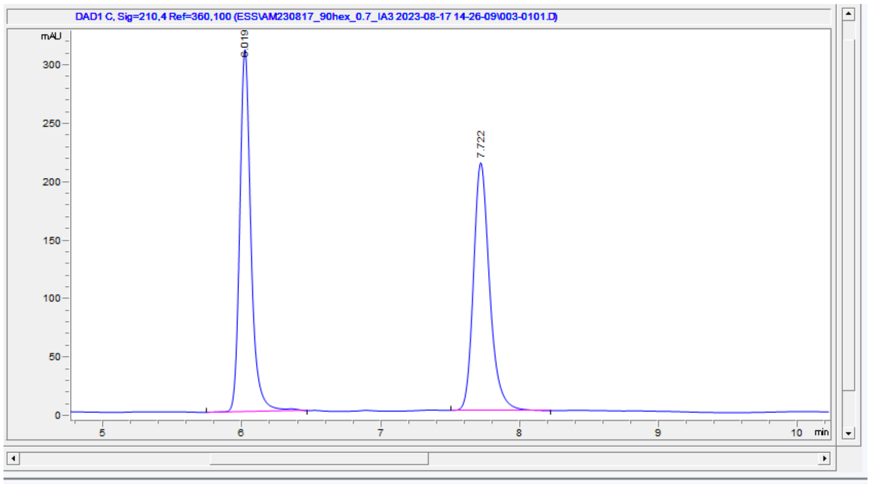

#	Time	Area	Height	Width	Area%	Symmetry
1	6.019	1743.7	311.1	0.0854	50.394	0.771
2	7.722	1716.4	212.2	0.1244	49.606	0.76

Figure S2. Chiral HPLC chromatogram of OMe-pCpd **7**.
HPLC conditions: (IA-3 column, 90:10 hexane/iPrOH, 0.700 mL/min)

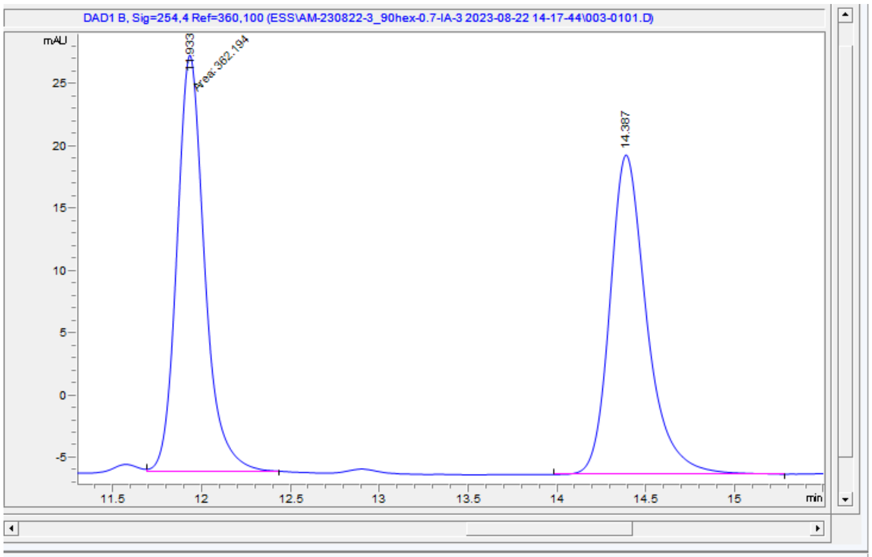

#	Time	Area	Height	Width	Area%	Symmetry
1	11.933	362.2	33.4	0.1805	49.514	0.841
2	14.387	369.3	25.6	0.2181	50.486	0.728

Figure S3. Chiral HPLC chromatogram of OH/OMe-pCpd **8**.
HPLC conditions: (IA-3 column, 90:10 hexane/iPrOH, 0.700 mL/min)

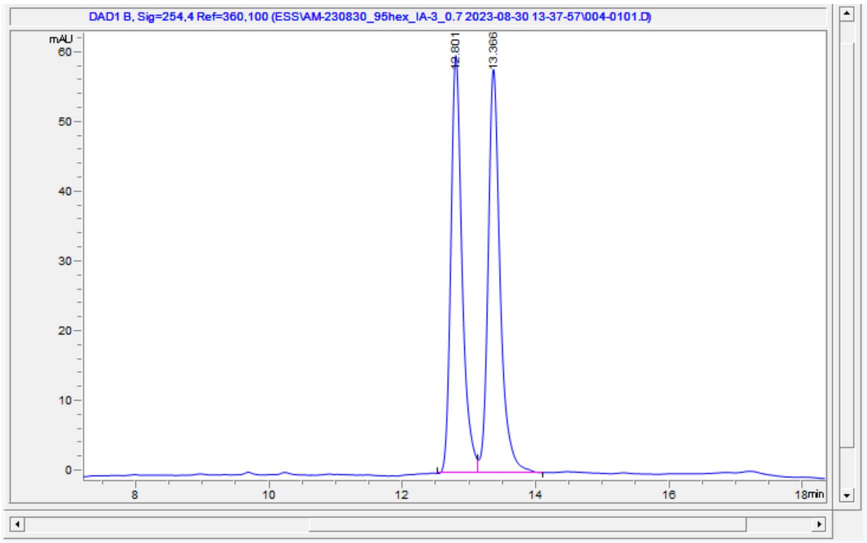

#	Time	Area	Height	Width	Area%	Symmetry
1	12.801	705.7	60.1	0.177	49.210	0.738
2	13.366	728.4	58.1	0.1863	50.790	0.736

Figure S4. Chiral HPLC chromatogram of quinone-pCpd **9**.
HPLC conditions: (IA-3 column, 95:5 hexane/iPrOH, 0.700 mL/min)

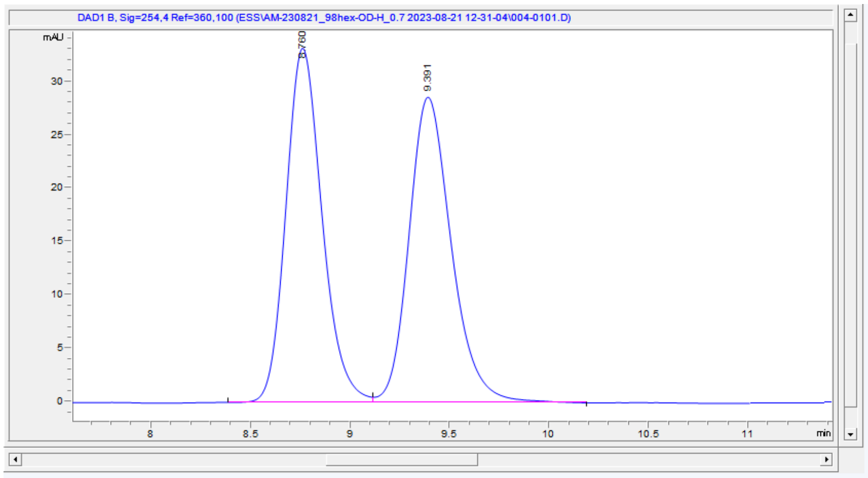

#	Time	Area	Height	Width	Area%	Symmetry
1	8.76	421.1	33.3	0.1957	49.715	0.815
2	9.391	425.9	28.7	0.2273	50.285	0.767

Figure S5. Chiral HPLC chromatogram of OctBr/OMe-pCpd **M1**.
HPLC conditions: (OD-H column, 98:2 hexane/iPrOH, 0.700 mL/min)

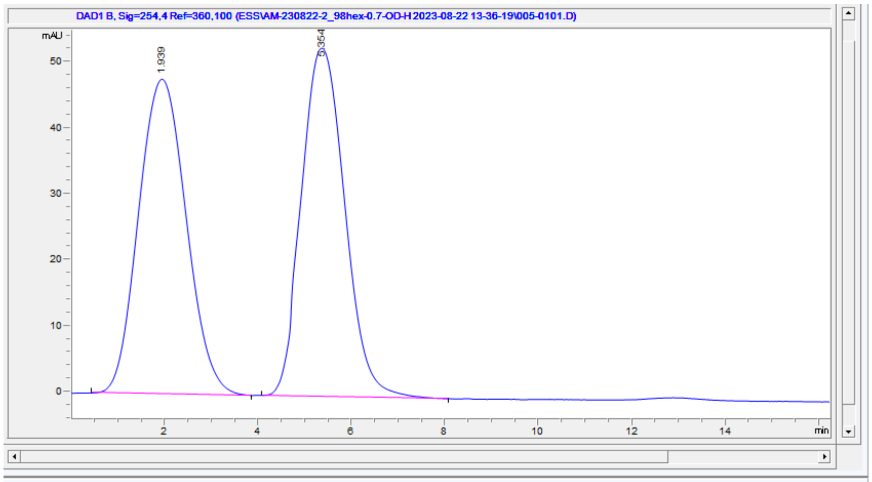

#	Time	Area	Height	Width	Area%	Symmetry
1	1.939	3376.6	47.8	1.085	49.754	0.925
2	5.354	3410	52.8	0.9914	50.246	0.842

Figure S6. Chiral HPLC chromatogram of TEG/OMe-pCpd **M2**.
HPLC conditions: (OD-H column, 98:2 hexane/iPrOH, 0.700 mL/min)

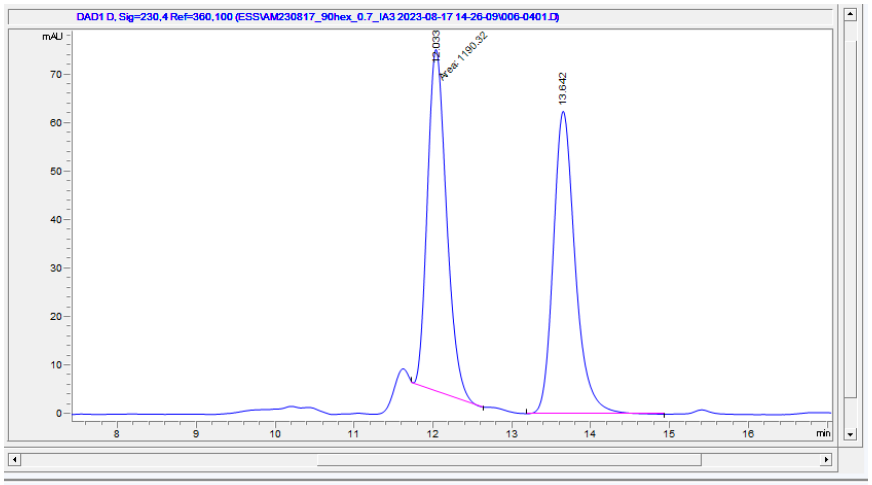

#	Time	Area	Height	Width	Area%	Symmetry
1	12.033	1190.3	70.6	0.281	49.656	0.774
2	13.642	1206.8	62.4	0.2936	50.344	0.763

Figure S7. Chiral HPLC chromatogram of OctAlaBoc/OMe-pCpd **M3**.
HPLC conditions: (IA-3 column, 90:10 hexane/iPrOH, 0.700 mL/min)

4. Polymerizations

4.1 *In-situ* ^{1}H NMR experiments

Into individual 1 dram vials, [2.2]paracyclophanediene monomer (0.063 mmol) and Grubbs' 3rd generation initiator (G3) were measured and brought into a nitrogen filled glovebox. A stock solution of G3 (0.019 M) was made in 0.45 mL of THF-d_8 and manually shaken for 2 minutes to fully dissolve the catalyst. The monomer was dissolved in 0.295 mL THF-d_8 and transferred to a J. Young NMR tube where 0.337 mL (0.0063 mmol G3) were added ([Monomer] = 100 mM). The NMR tube was sealed, removed from the glovebox, wrapped in aluminum foil, and placed in an ice bath. The sample was removed from the aluminum foil, whipped with a kimwipe, placed into a 600 MHz NMR at 25 ˚C and the first ^{1}H NMR spectrum was recorded (t = 0 min). The spectrometer was then heated to 50 ˚C and ^{1}H NMR spectra were recorded every 5 minutes for the first hour and then every 10 minutes until the polymerization was complete.

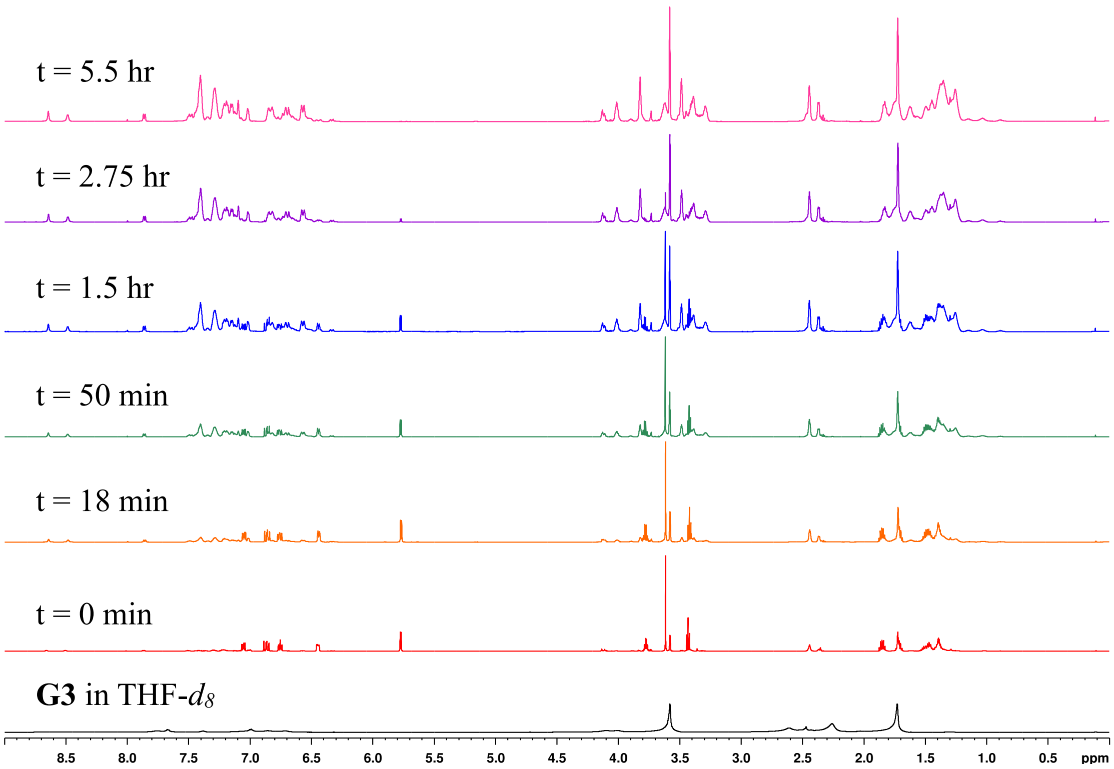

Figure S8. *In-situ* ^{1}H NMR experiment monitoring the ROMP of monomer OctBr/OMe-pCpd **M1** with G3 at 50 ˚C in THF-d_8 showing monomer consumption over time.

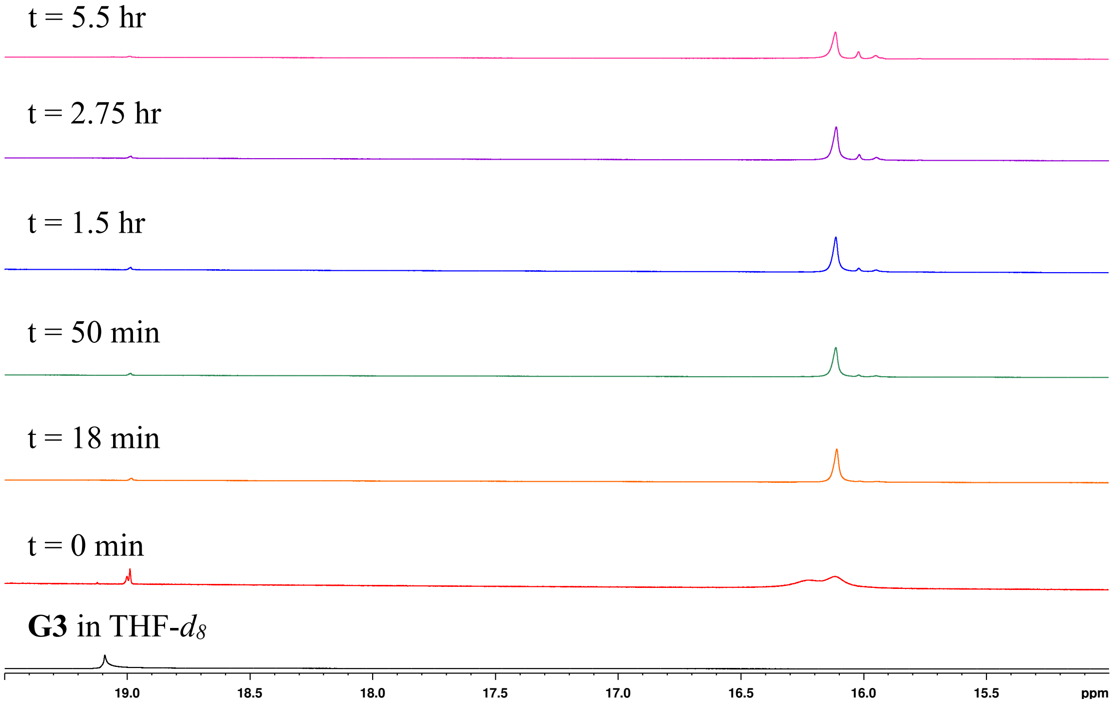

Figure S9. *In-situ* ^{1}H NMR experiment monitoring the ROMP of monomer OctBr/OMe-pCpd **M1** with G3 at 50 ˚C in THF-d_8 showing shifts in the carbene signal over time.

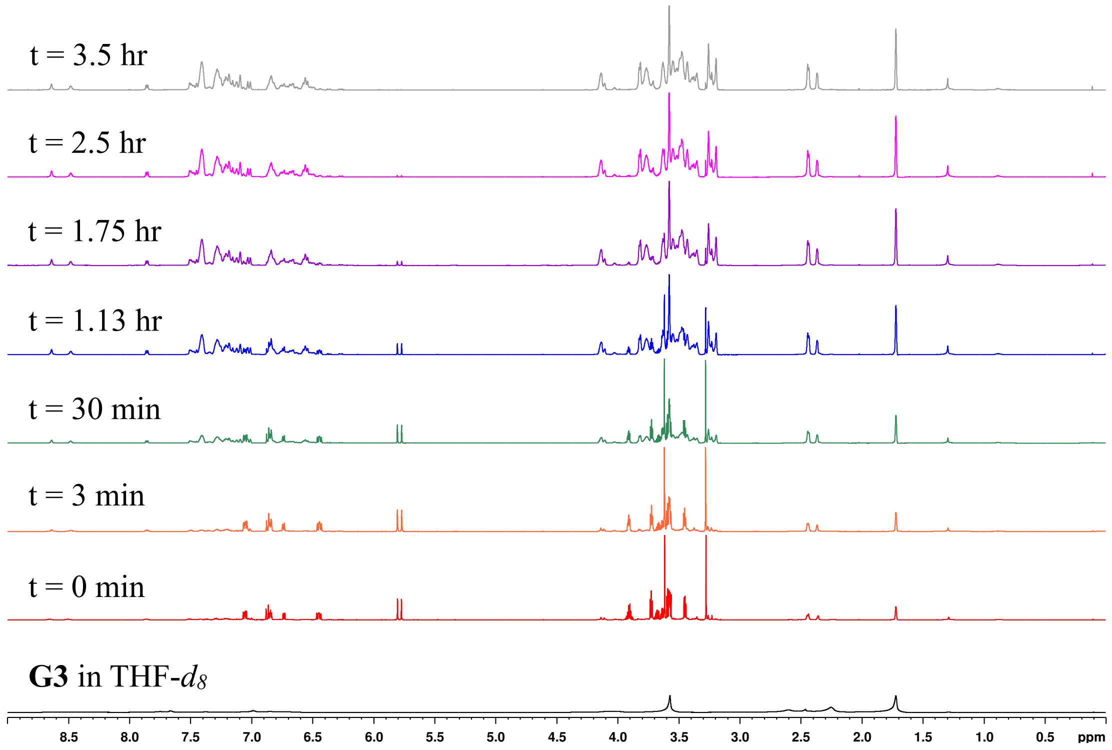

Figure S10. *In-situ* ^{1}H NMR experiment monitoring the ROMP of monomer TEG/OMe-pCpd **M2** with G3 at 50 ˚C in THF-d_8 showing monomer consumption over time.

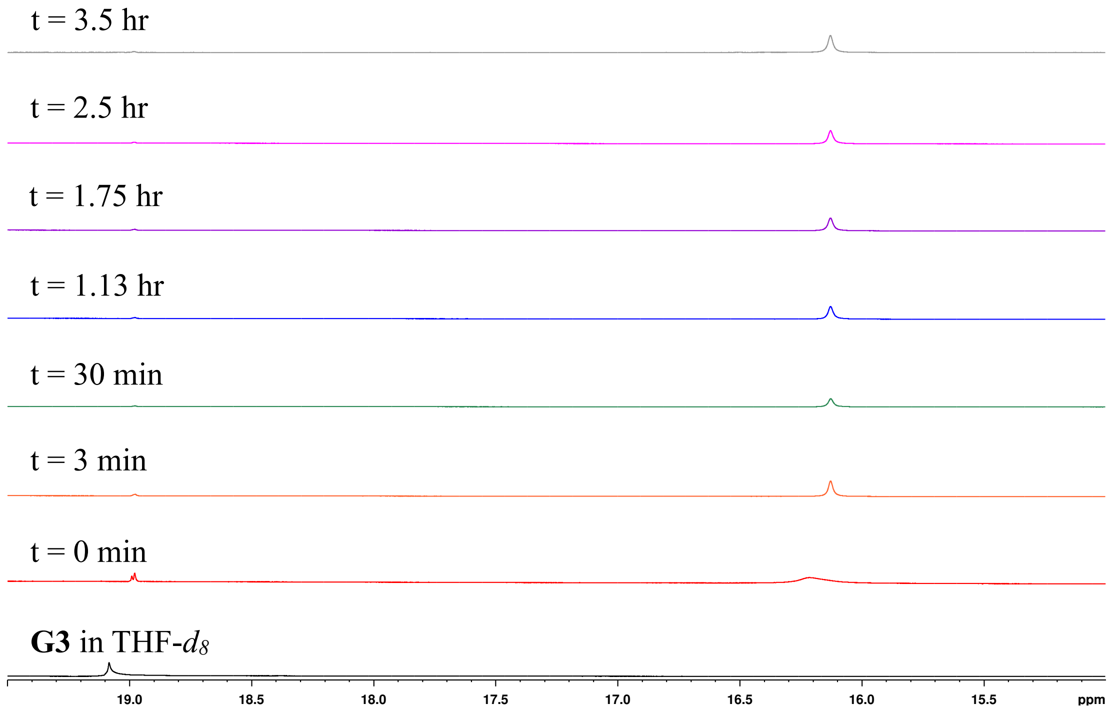

Figure S11. *In-situ* ^{1}H NMR experiment monitoring the ROMP of monomer TEG/OMe-pCpd **M2** with G3 at 50 ˚C in THF-d_8 showing shifts in the carbene signal over time.

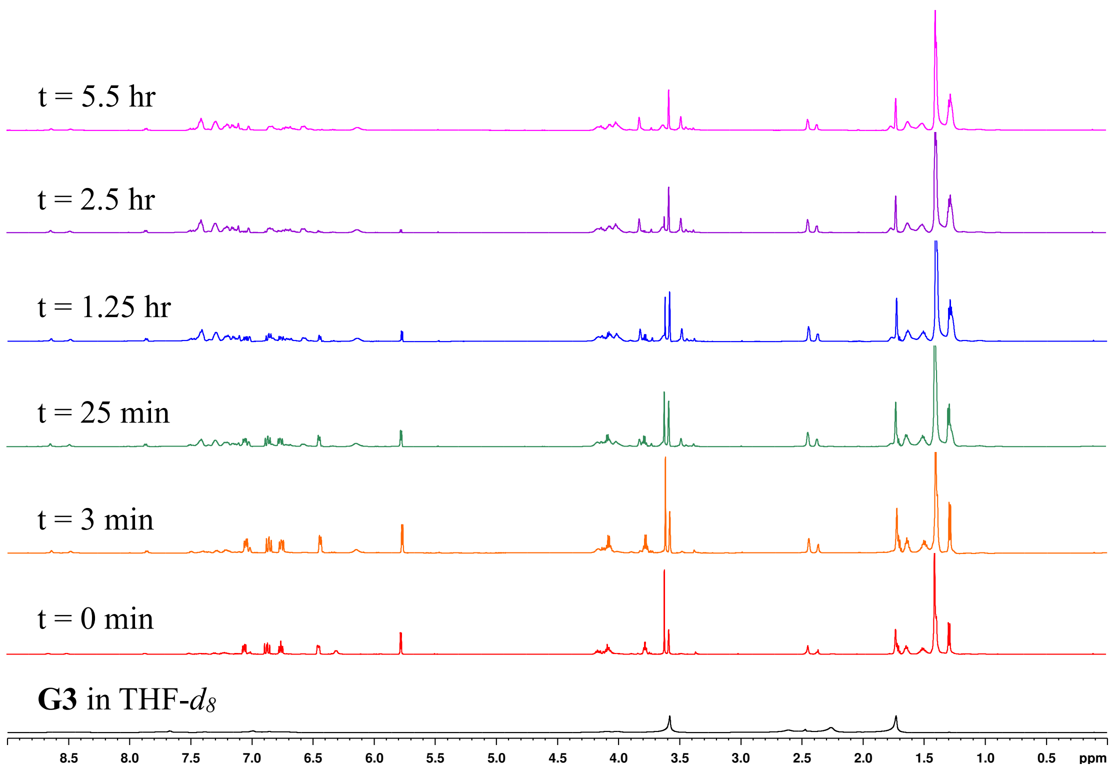

Figure S12. *In-situ* ^{1}H NMR experiment monitoring the ROMP of monomer OctAlaBoc/OMe-pCpd **M3** with G3 at 50 ˚C in THF-d_8 showing monomer consumption over time.

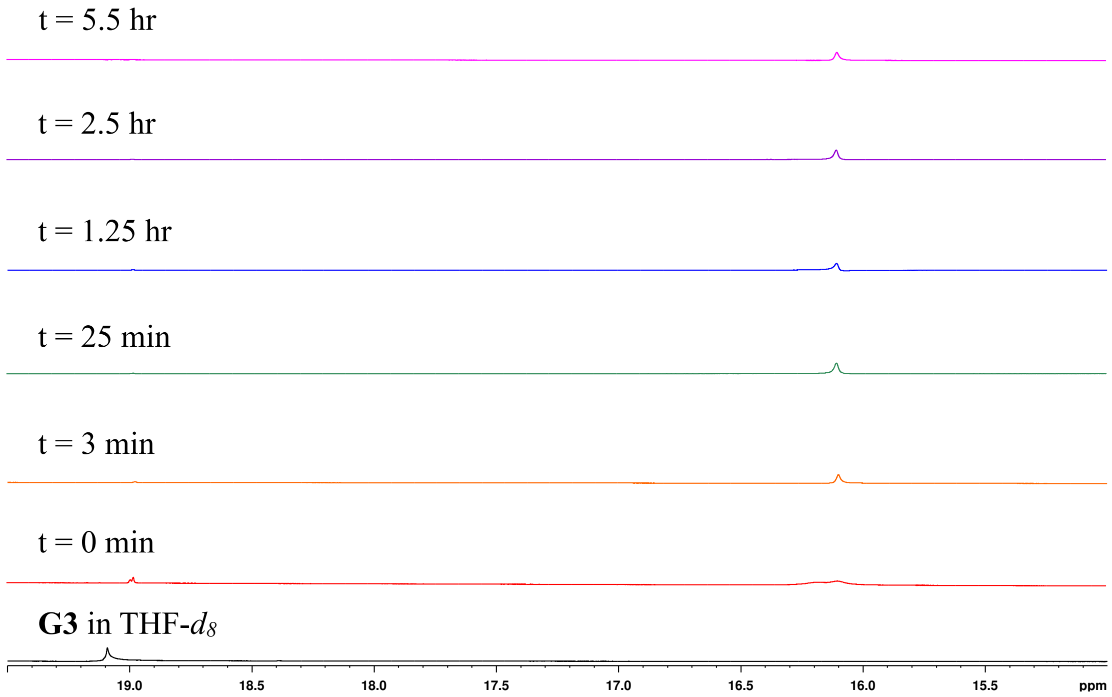

Figure S13. *In-situ* ^{1}H NMR experiment monitoring the ROMP of monomer OctAlaBoc/OMe-pCpd **M3** with G3 at 50 ˚C in THF-d_8 showing shifts in the carbene signal over time.

4.2 ROMP of monomers with G3

General polymerization procedure: In a nitrogen filled glovebox, a stock solution of G3 initiator (10 mol %) was prepared in anhydrous, degassed THF. Cyclophanediene monomer (25 mg) was weighed out into 1 dram vials and brought into a nitrogen filled glovebox. The monomer was dissolved in degassed THF and transferred to a Schlenk tube with a stir bar. An appropriate amount of the G3 solution was added for the desired equivalence of monomer-to-catalyst ratio (20, 25, or 30) and for [monomer] = 100 mM. The Schlenk tube was sealed, removed from the glovebox, and wrapped in aluminum foil to be placed in an oil bath at 50 ˚C and stirred until the monomer was completely consumed based off the in-situ NMR experiments (**M2**: 21 min/monomer, **M1** and **M3**: 33 min/monomer). The reaction was cooled to room temperature, degassed ethyl vinyl ether (0.6 mL) was added under an inert atmosphere and the mixture was allowed to stir for at least 12 hrs. The reaction was then opened to air and the polymer was precipitated by the addition of 4 mL of cold methanol. A pipette Celite column was run washing with methanol followed by dissolving the polymer with dichloromethane collected into a separate scintillation vial. The DCM layer was evaporated under reduced pressure in the dark to give the desired polymer as an orange film.

The 10 mer was synthesized during the *in-situ* ^{1}H NMR kinetics experiments. When the monomer was seen to be completely consumed by NMR spectroscopy, the J. Young tube was removed from the spectrometer, placed in aluminum foil, and returned to the glove box. Degassed ethyl vinyl ether (0.6 mL) was added to the J. Young tube where it was sealed, inverted once, and removed from the box. The sealed NMR tube was placed on a shaker and left for at least 12 hrs. The polymer was then precipitated and filtered the same as the other samples.

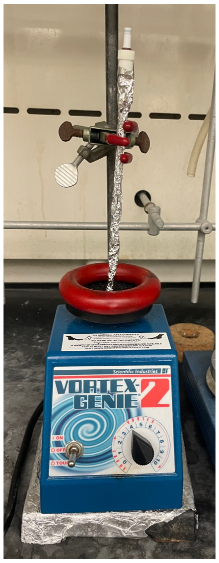

Figure S14. Set up of the J. Young NMR tube wrapped in aluminum foil placed on a shaking plate.

4.3 Photoisomerization

General procedure: 10 mg of polymer (30 mer) was dissolved in DCM-d_2 (0.75 mL) and added into a NewEra PhotoNMR tube and wrapped in aluminum foil until it was placed in a 400 MHz NMR spectrometer. An optical cable (395 nm) inserted into the inner chamber of the photoNMR tube and the sample was manually inserted into the NMR spectrometer. Before turning on the

optical cable, a ^{1}H NMR spectrum was collected (t = 0 min). The optical cable was then turned on and allowed to irradiate the sample in the NMR spectrometer with ^{1}H NMR spectra being collected. When the sample was fully isomerized and removed from the NMR spectrometer, the sample was visibly more orange with orange solids. The sample was poured into a scintillation vial and the solvent was removed under reduced pressure to a dark orange solid.

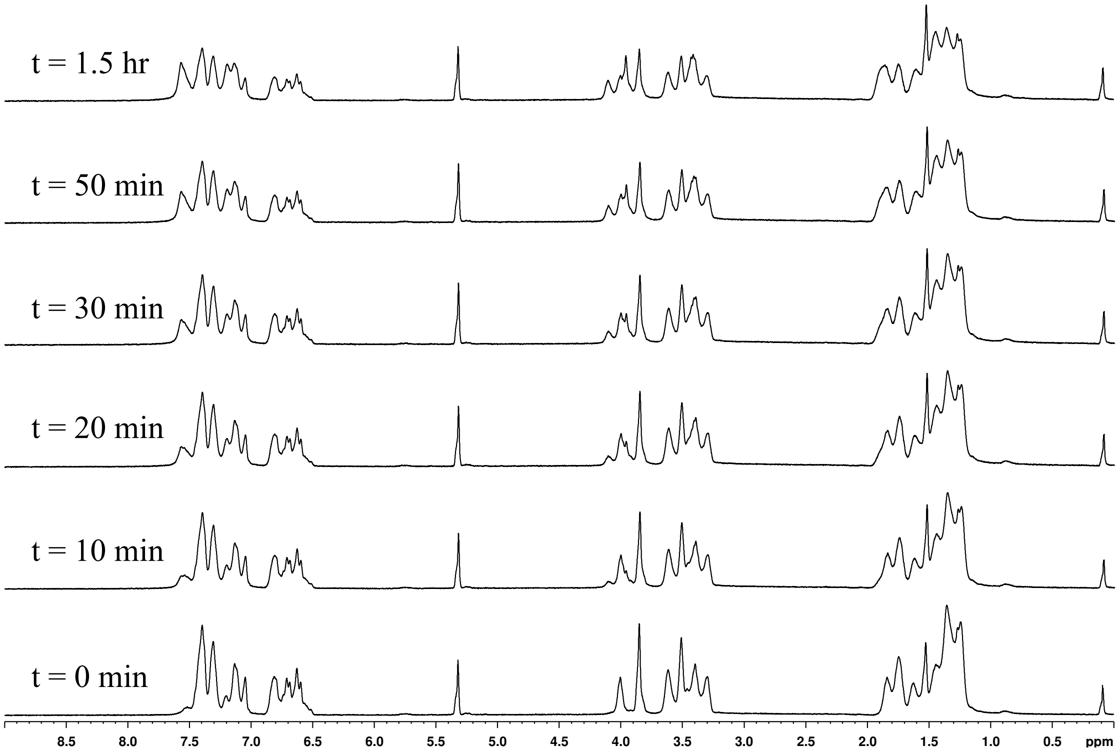

Figure S15. PhotoNMR spectroscopy experiment tracking the photoisomerization of polymer OctBr/OMe-PPV 30 mer **P1d** by ^{1}H NMR spectroscopy irradiating at 395 nm in DCM-d_2. The peaks of the conjugated backbone (δ 7.7-6.4 ppm) converge upfield while the methylene groups attached to the oxygen atom on PPV's backbone significantly decreased around δ 3.5 ppm while the *trans* stereoisomer peak around δ 4.2 ppm becomes more prominent.

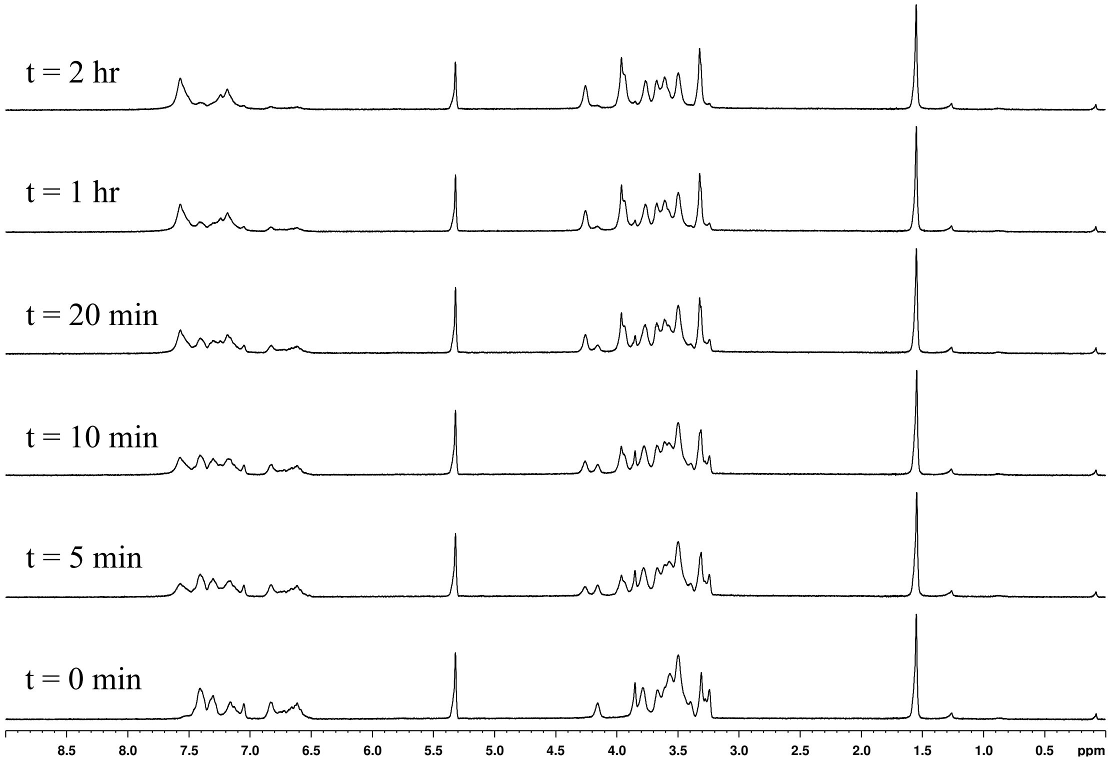

Figure S16. PhotoNMR spectroscopy experiment tracking the photoisomerization of polymer TEG/OMe 30 mer **P2d** by ^{1}H NMR spectroscopy irradiating at 395 nm in DCM-d_2. The peaks of the conjugated backbone (δ 7.7-6.4 ppm) converge upfield while the methylene groups attached to the oxygen atom on PPV's backbone significantly decreased around δ 3.5 ppm while the *trans* stereoisomer peak around δ 4.2 ppm becomes more prominent.

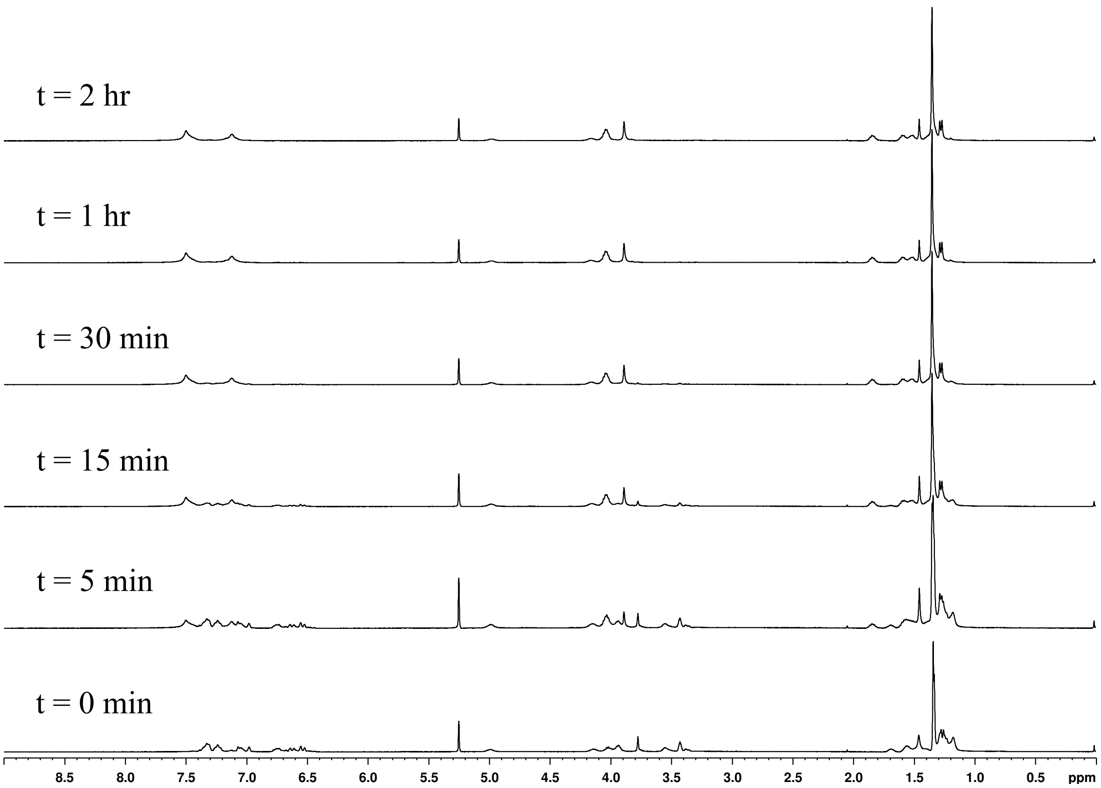

Figure S17. PhotoNMR spectroscopy experiment tracking the photoisomerization of polymer OctAlaBoc/OMe 30 mer **P3d** by ^{1}H NMR spectroscopy irradiating at 395 nm in DCM-d_2. The peaks of the conjugated backbone (δ 7.7-6.4 ppm) converge upfield while the methylene groups attached to the oxygen atom on PPV's backbone disappears around δ 3.5 ppm while the *trans* stereoisomer peak around δ 4.2 ppm becomes more prominent.

4.4 Polymerization of Diblock Copolymers

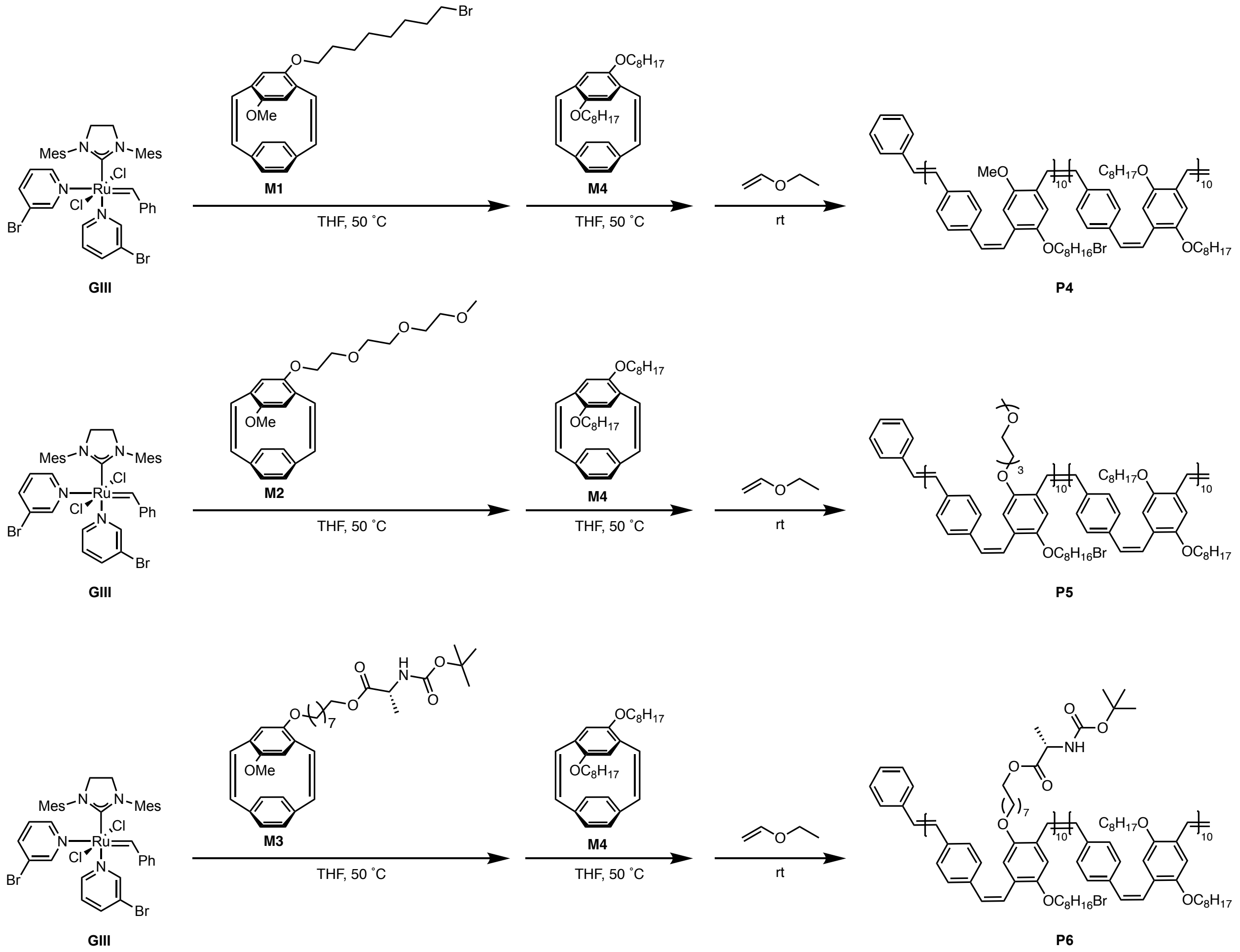

Scheme S1. Synthetic scheme for PPV diblocks.

In a nitrogen filled glovebox, a stock solution of G3 initiator (10 mol %) was prepared in anhydrous, degassed THF. Each of the cyclophanediene monomer (25 mg) was individually weighed out into a 1 dram vial and brought into a nitrogen filled glovebox. The monomer was dissolved in degassed THF and transferred to a Schlenk tube with a stir bar. An appropriate amount of the G3 solution was added for the desired equivalence of monomer-to-catalyst ratio (10:1) and for [monomer] = 100 mM. The Schlenk tube was sealed, removed from the glovebox, and wrapped in aluminum foil to be placed in an oil bath at 50 ˚C and stirred until the monomer was completely consumed based off the experimentally determined time from the in-situ NMR experiments (TEG/OMe: 21 min/monomer, OctBr/OMe and OctAlaBoc/OMe: 33 min/monomer). The reaction was cooled to room temperature and returned to the glovebox and an aliquot was taken for GPC. A stock solution of 4,7-dioctyloxy-[2.2]paracyclophane-1,9-diene (100 mM) was prepared in THF and the appropriate amount was added to each polymerization for the addition of a 10mer. The resealed Schlenk tube in aluminum foil was removed from the glovebox and heated at 50 ˚C for 6 hours. After this time the polymerizations were cooled to room temperature and degassed ethyl vinyl ether (0.6 mL) was added under an inert atmosphere and stirred for at least 12 hrs. The reaction was then opened to air and the polymer was precipitated by the addition of 4 mL of cold methanol. A pipette Celite column was run washing with methanol followed by dissolving the

polymer with dichloromethane collected into a separate scintillation vial. The DCM layer was evaporated under reduced pressure in the dark to give the desired polymer as orange solids.

Table S1. Synthesis, GPC data, and optical characterization of PPV diblocks **P4**, **P5**, and **P6**.[a] M_n GPC values were determined against polystyrene standards. [b] Measurements were done in dilute solutions of chloroform.

Diblock Copolymer	**P4**	**P5**	**P6**
First pCpd measured (mg)	26.5 of **M1**	25.1 of **M2**	28.2 of **M3**
Amount of G3 added (mg)	5.3	5.6	4.5
Total THF added with first block (mL)	0.600	0.635	0.514
p-dioctyloxy-pCpd (**M4**) added (mg)	27.6	29.2	23.7
THF added with second block (mL)	0.600	0.634	0.514
Yield (%)	93	77	88
Dispersity[a]	1.18	1.15	1.15
Absorbance max[b]	442	428	428
Emission max[b]	523	523	523

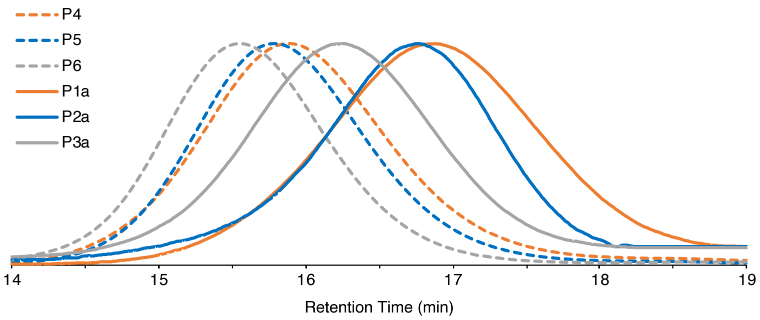

Figure S18. GPC chromatogram of block extension study showing the change in retention size to their monoblock (solid) compared to the diblock polymers (dashed). GPC in THF using the RI detector.

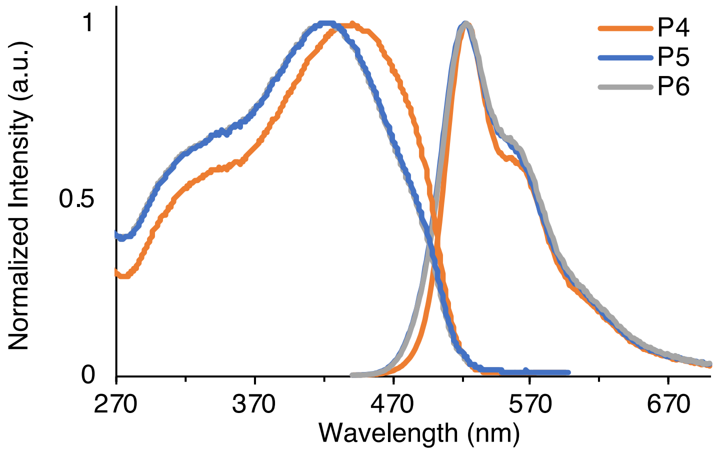

Figure S19. Absorbance and emission spectra of PPV polymers P4-6.

4.5 Post-polymerization modification

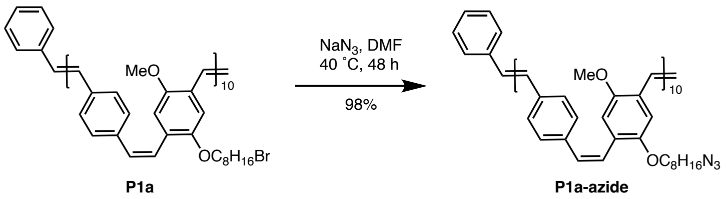

P1a-azide:

PPV-Br **P1a** (20 mg) and sodium azide (14.3 mg) were mixed in 4.4 mL of DMF and were stirred at 40 ˚C for 48 hours. The mixture was extracted using chloroform (30 mL x 3) and brine. The combined organic phases were dried using Na_2SO_4. The solvent was removed under reduced pressure. The crude product was dissolved in minimal amount of THF and precipitated in cold methanol. The product was collected by filtration as a red solid (18 mg, Mn 5.1 kDa, PDI 1.28, yield 98%).

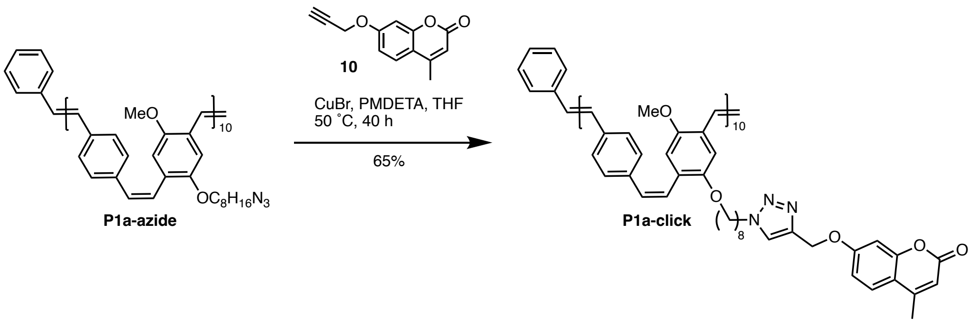

P1a-click:

P1a-azide (18 mg), alkyne-coumarin **10** (19 mg, 0.088 mmol), CuBr (31 mg, 0.22 mmol), and PMDETA (38 mg, 0.22 mmol) were dissolved in 4 mL of THF. The mixture was degassed by three freeze-pump-thaw procedures and refilled with argon. The mixture was heated to 50 ºC and stirred for 40 hours. The mixture was passed through a neutral alumina column to remove the copper salt. The collected mixture was condensed and precipitated in cold methanol. The product was collected by filtration as a red solid (18 mg, yield 65%, Mn 6.0 kDa, PDI 1.24).

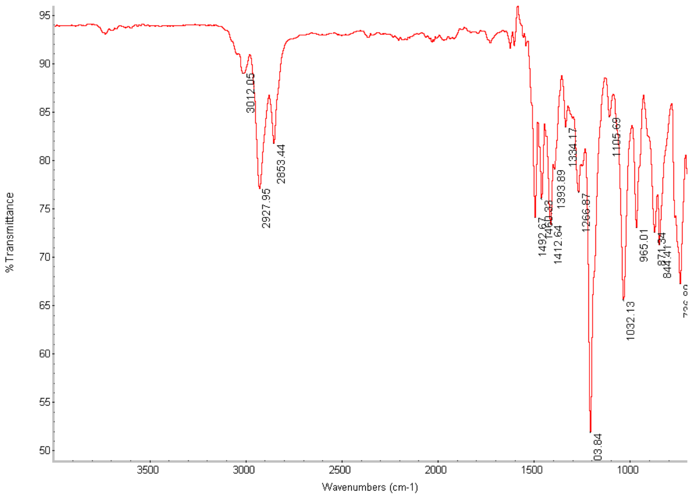

Figure S20. FT-IR spectrum of **P1a**.

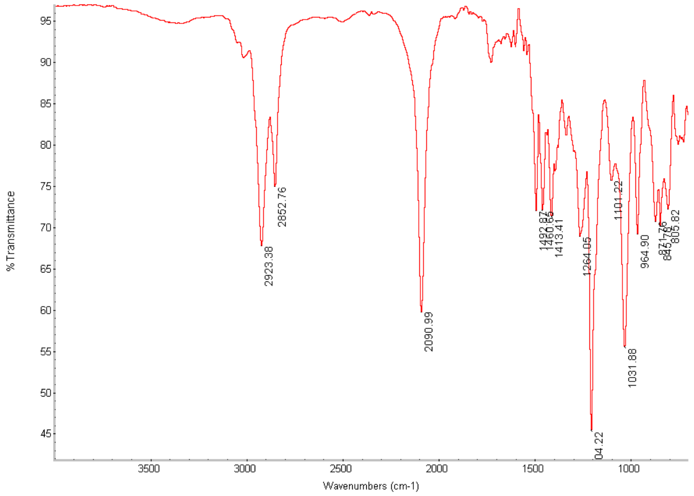

Figure S21. FT-IR spectrum of **P1a-azide**.

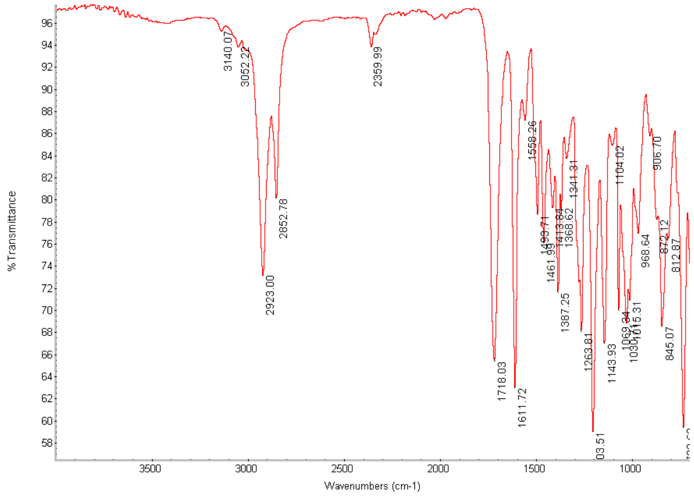

Figure S22. FT-IR spectrum of **P1a-click**.

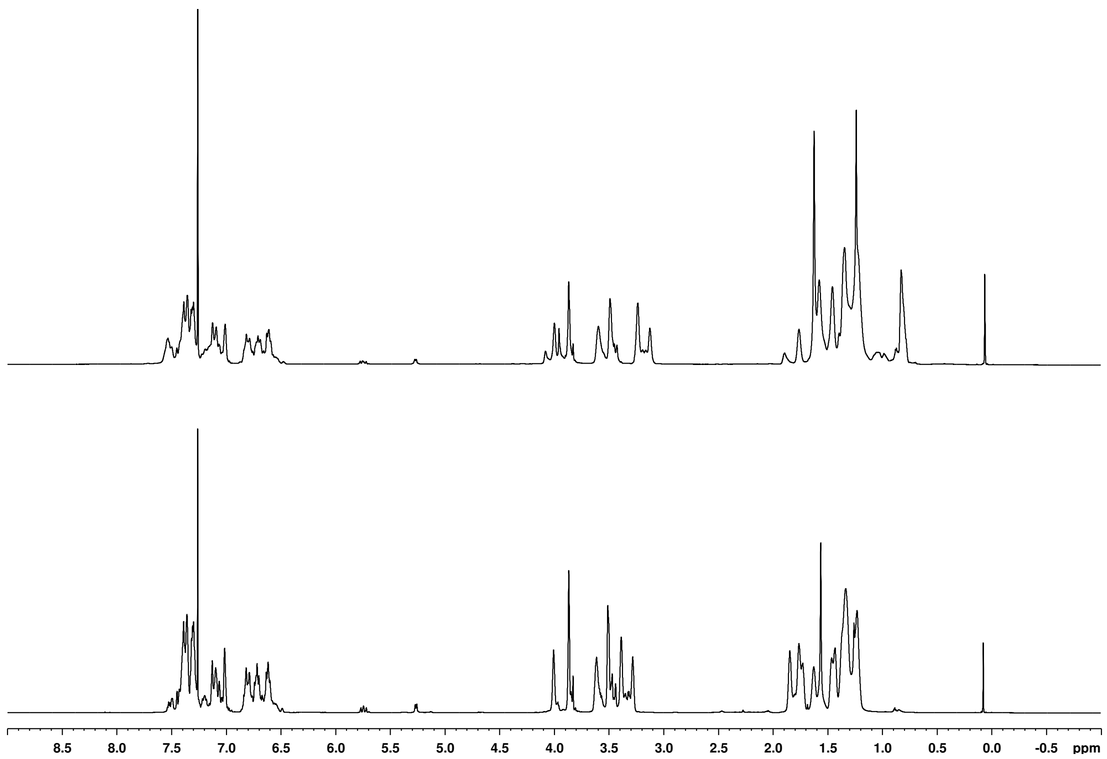

Figure S23. ^{1}H NMR spectrums of the bromide containing **P1a** (bottom) and **P1a-azide** (top).

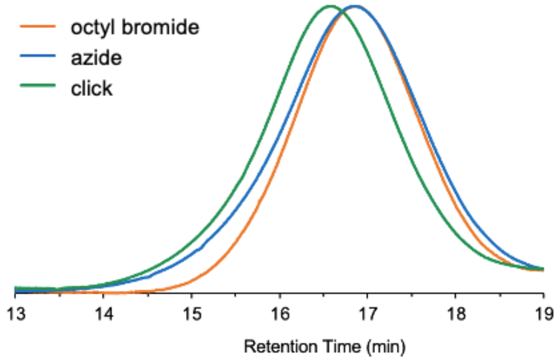

Figure S24. GPC chromatogram of post polymerization modification for bromine-azide exchange (**P1a** to **P1a-azide**) and azide-alkyne click reaction (**P1a-click**). GPC in THF using the RI detector.

Table S2. GPC data and optical characterization of PPVs. [a] calculated based on the targeted degree of polymerization noted in [M]/[GIII]. [b] M_n GPC values were determined against polystyrene standards. [c] Measurements were done in dilute solutions of chloroform.

PPV	$M_{n,\ calc}$[a]	$M_{n,\ GPC}$[b]	Đ	% yield	abs. λmax (nm)[c]	em. λmax (nm)[c]
**P1a-azide**	4139	5100	1.27	98	437	523
**P1a-click**	6281	6000	1.23	65	446	523

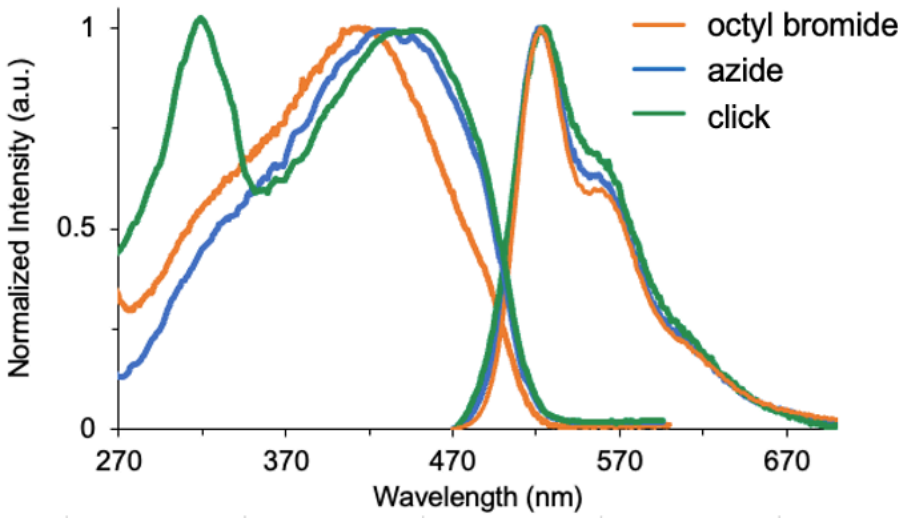

Figure S25. Absorbance and emission spectra of PPV polymers **P1a**, **P1a-azide**, and **P1a-click**.

4.6 MALDI-TOF-MS

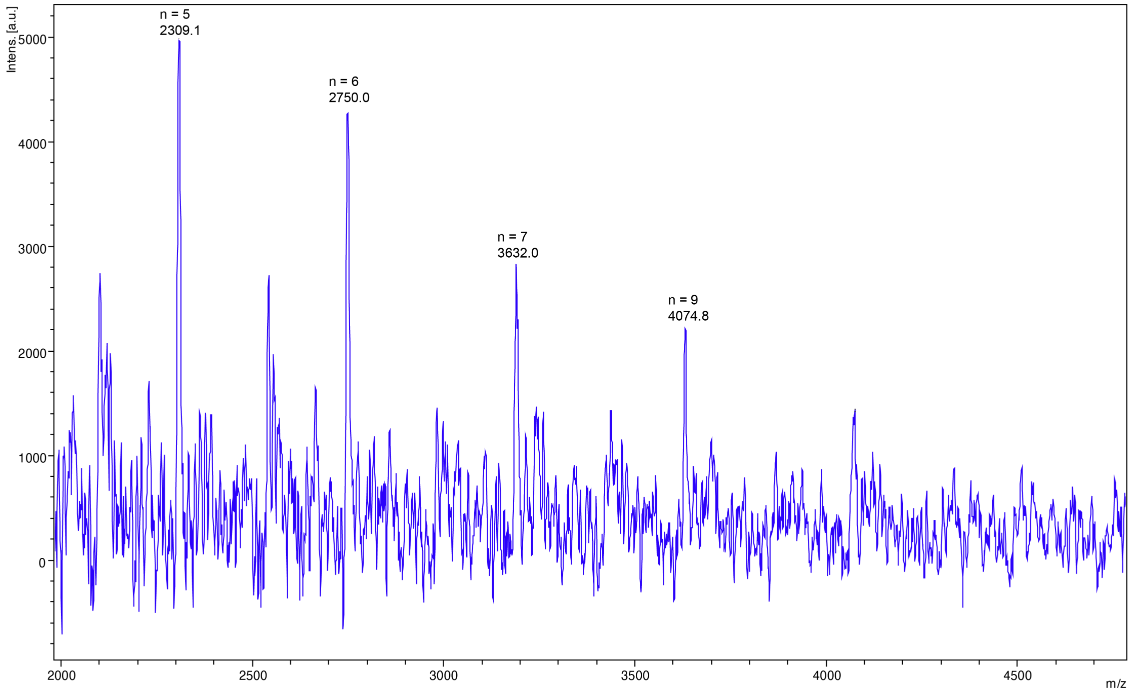

Figure S26. MALDI-TOF spectra of OctBr-PPV 10 mer **P1a**. The observed mass shift of 441 amu agrees with the calculated mass of the octBr-pCpd monomer.

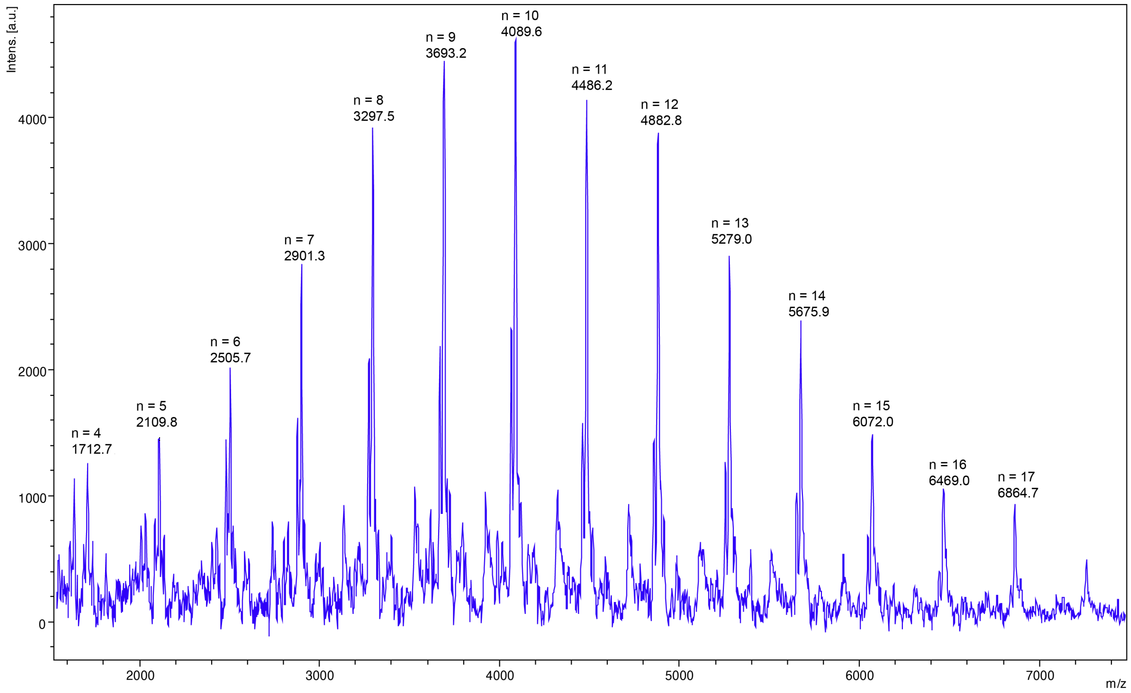

Figure S27. MALDI-TOF spectra of TEG-PPV 10 mer **P2a**. The observed mass shift of 396 amu agrees with the calculated mass of the TEG-pCpd monomer. The two major series of peaks are consistent with the vinyl end group (minor series) and the sodium adduct (major series).

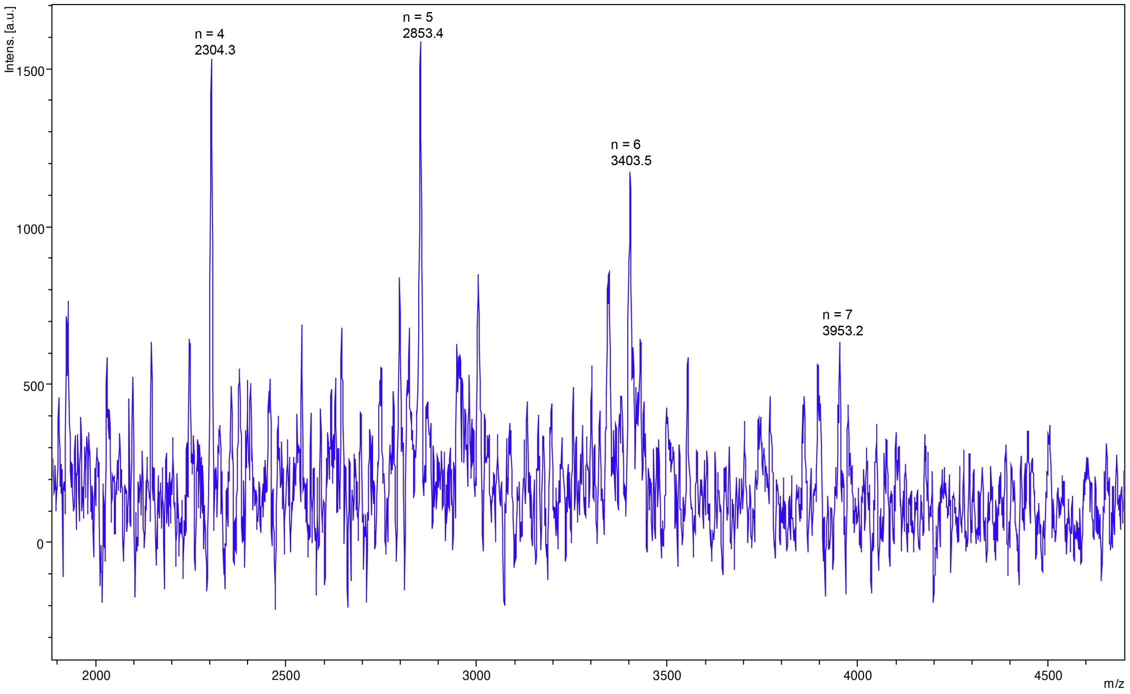

Figure S28. MALDI-TOF spectra of OctAlaBoc-PPV 10 mer **P3a**. The observed mass shift of 550 amu agrees with the calculated mass of the OctAlaBoc-pCpd monomer.

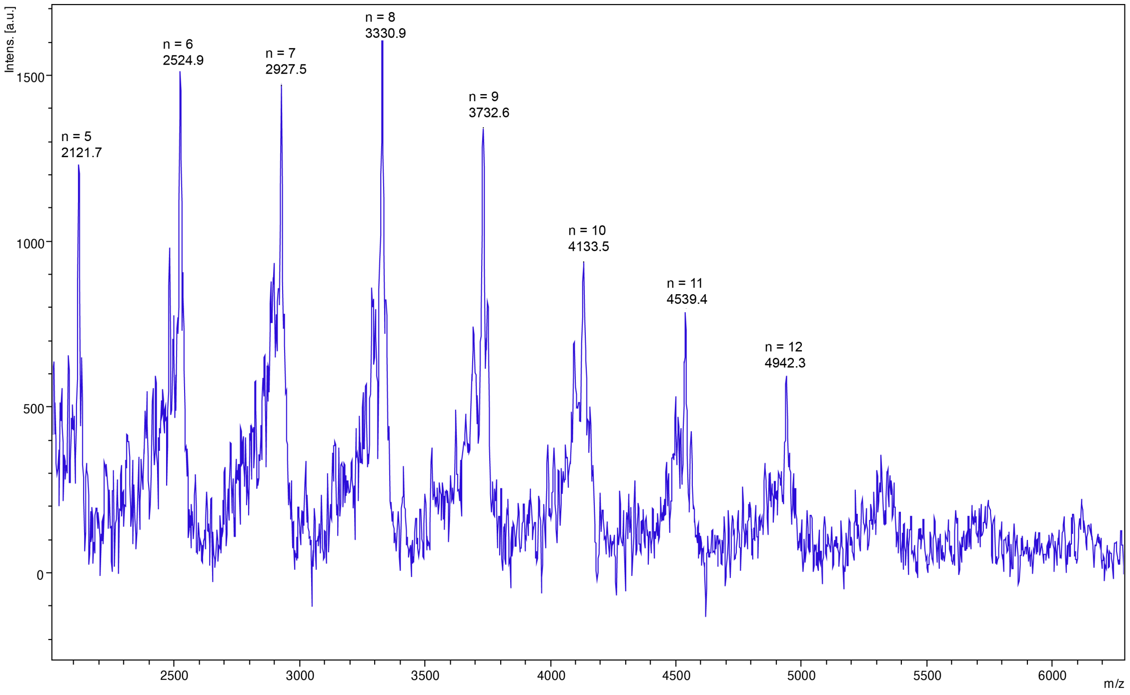

Figure S29. MALDI-TOF spectra of Azide-PPV 10 mer **P1a-azide**. The observed mass shift of 403 amu agrees with the calculated mass of replacing a bromide with an azide.

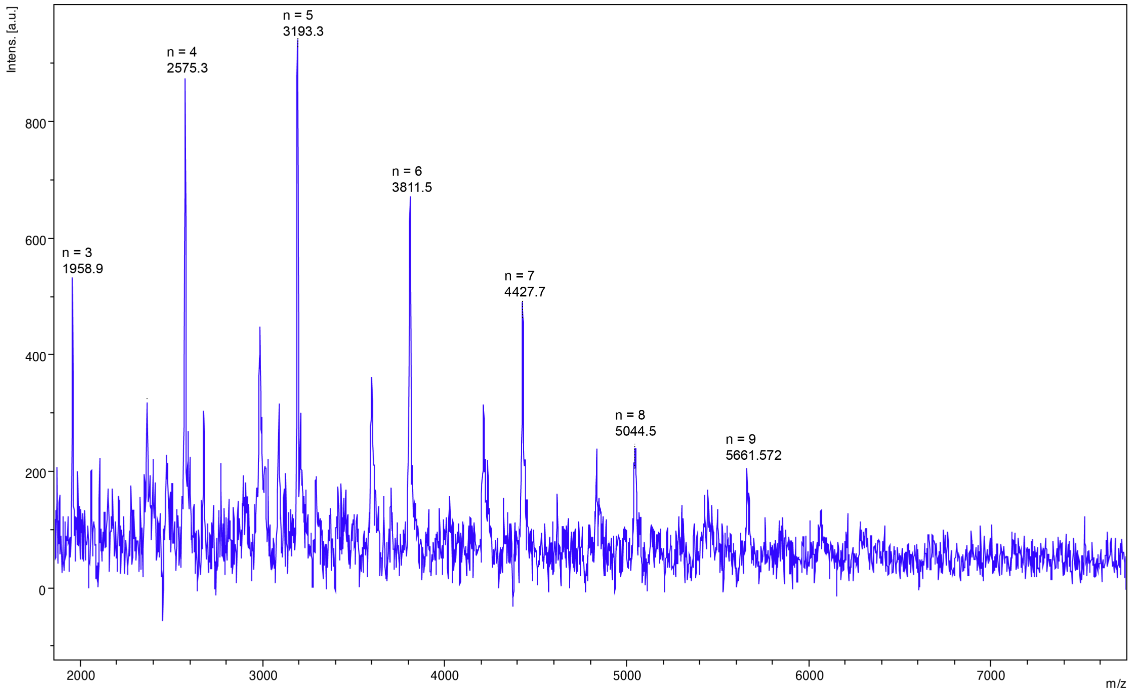

Figure S30. MALDI-TOF spectra of coumarin-click-PPV 10 mer **P1a-click**. The observed mass shift of 617 amu agrees with the calculated mass of the post-polymerization click reaction between the azide and coumarin alkyne **10**.

5. References

[1] Shin, S., Menk, F., Kim, Y., Lim, J., Char, K., Zentel, R., Choi, T. L. Living Light-Induced Crystallization-Driven Self-Assembly for Rapid Preparation of Semiconducting Nanofibers. *J. Am. Chem. Soc.* **2018**, *140*, 6088-6094.

[2] Elacqua, E., Manning, K. B., Lye, D. S., Pomarico, S. K., Morgia, F., Weck, M. Supramolecular Multiblock Copolymers Featuring Complex Secondary Structures. *J. Am. Chem. Soc.* **2017**, *139*, 12240-12250.

[3] Rull-Barrull, J., d'Halluin, M., Le Grognec, E., Felpin, F.-X. Harnessing the Dual Properties of Thiol-Grafted Cellulose Paper for Click Reactions: A Powerful Reducing Agent and Adsorbent for Cu. *Angew. Chem. Int. Ed.* **2016**, *55*, 13549-13552.

6. NMR Spectra

^{1}H NMR spectrum of **7**

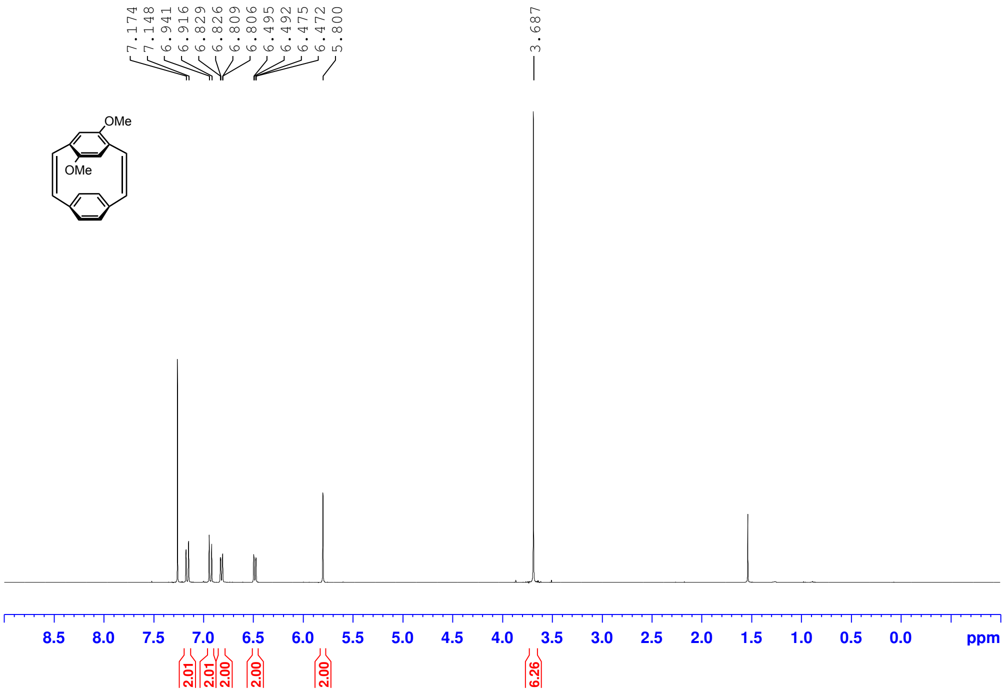

^{13}C NMR spectrum of **7**

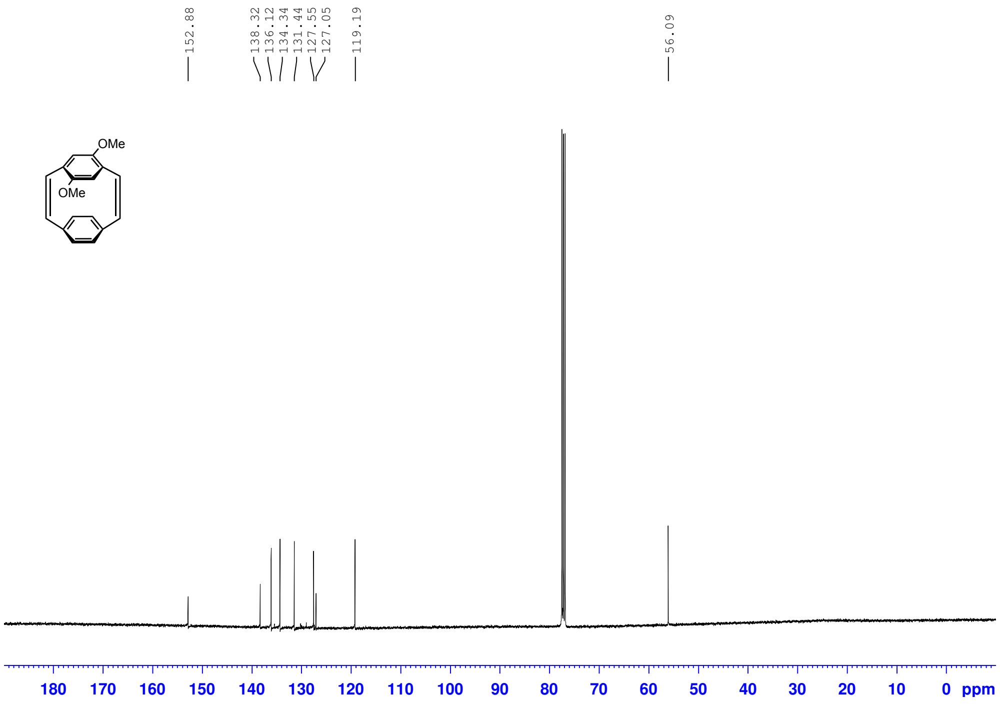

^{1}H NMR spectrum of **M4**

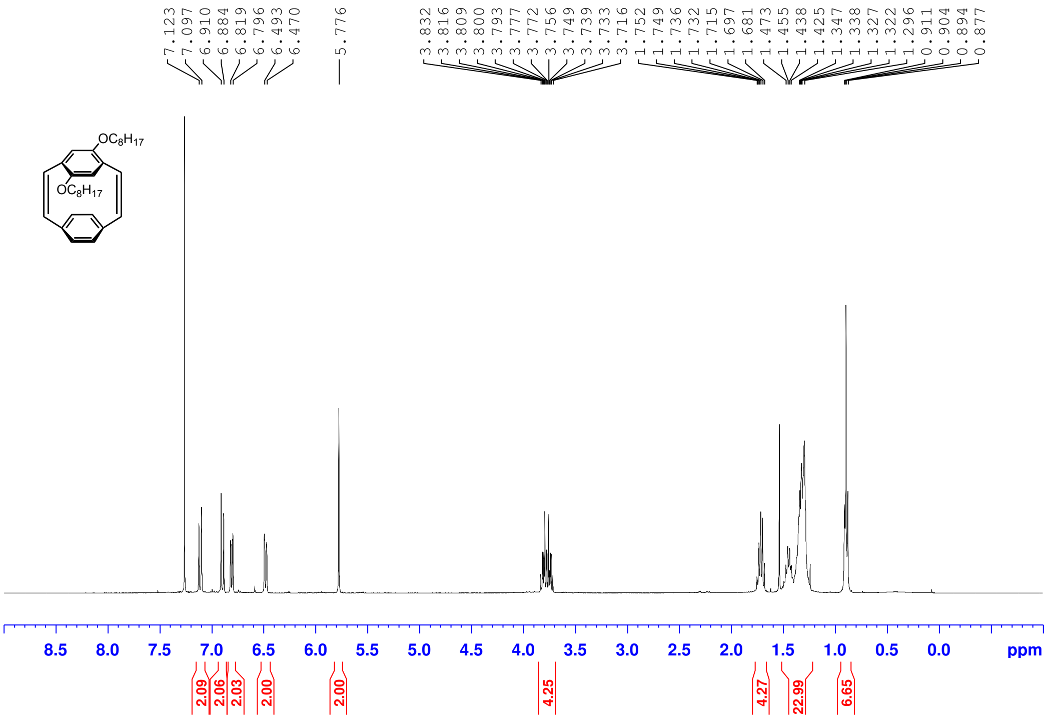

^{1}H NMR spectrum of **10**

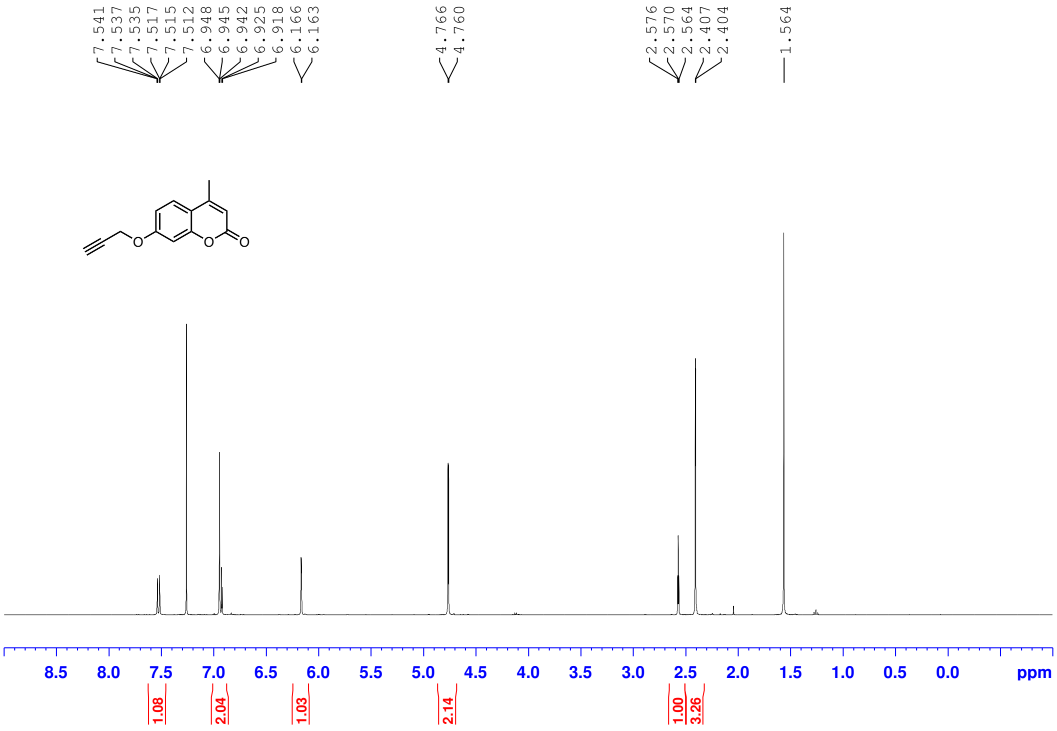

^{1}H NMR spectrum of **8**

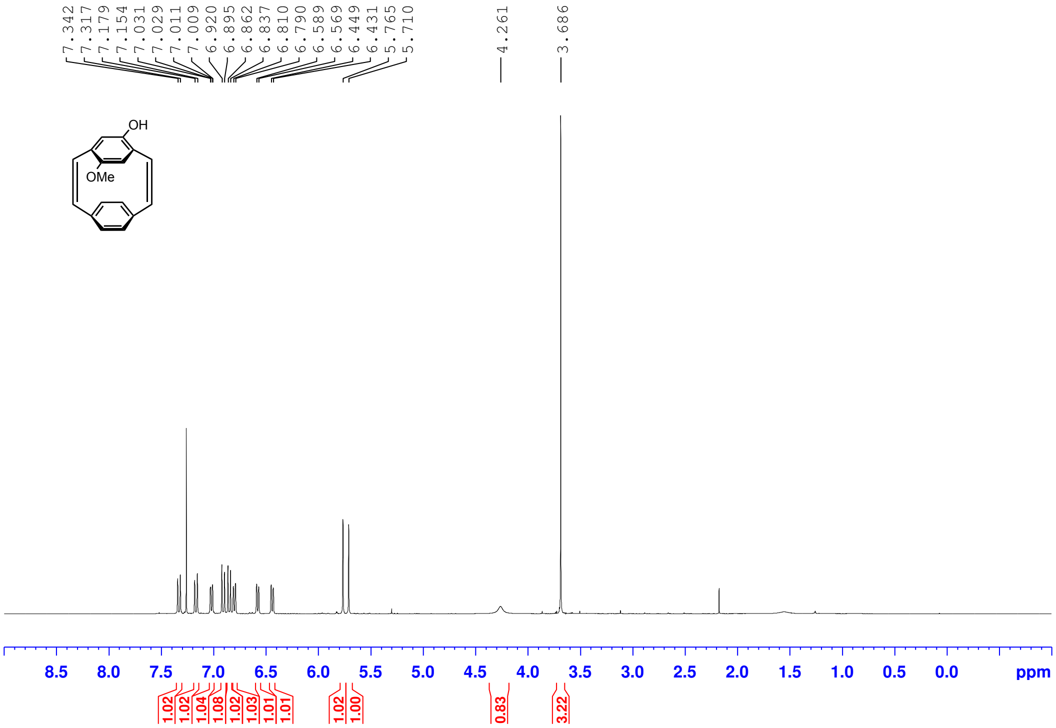

^{13}C NMR spectrum of **8**

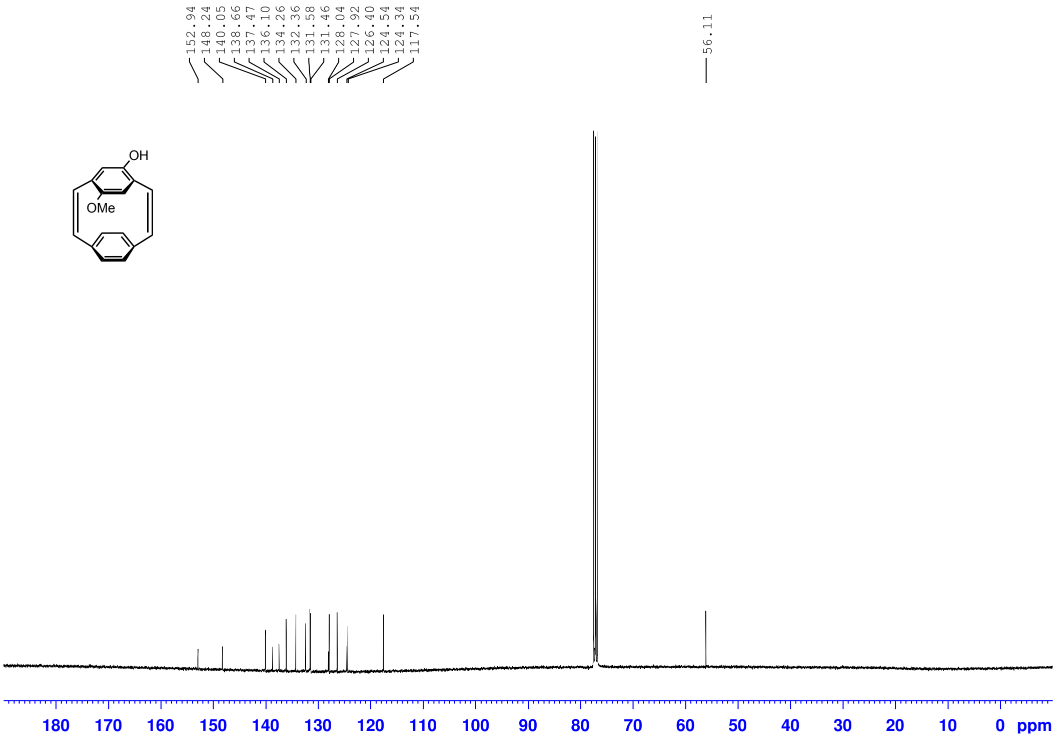

HSQC of **8**

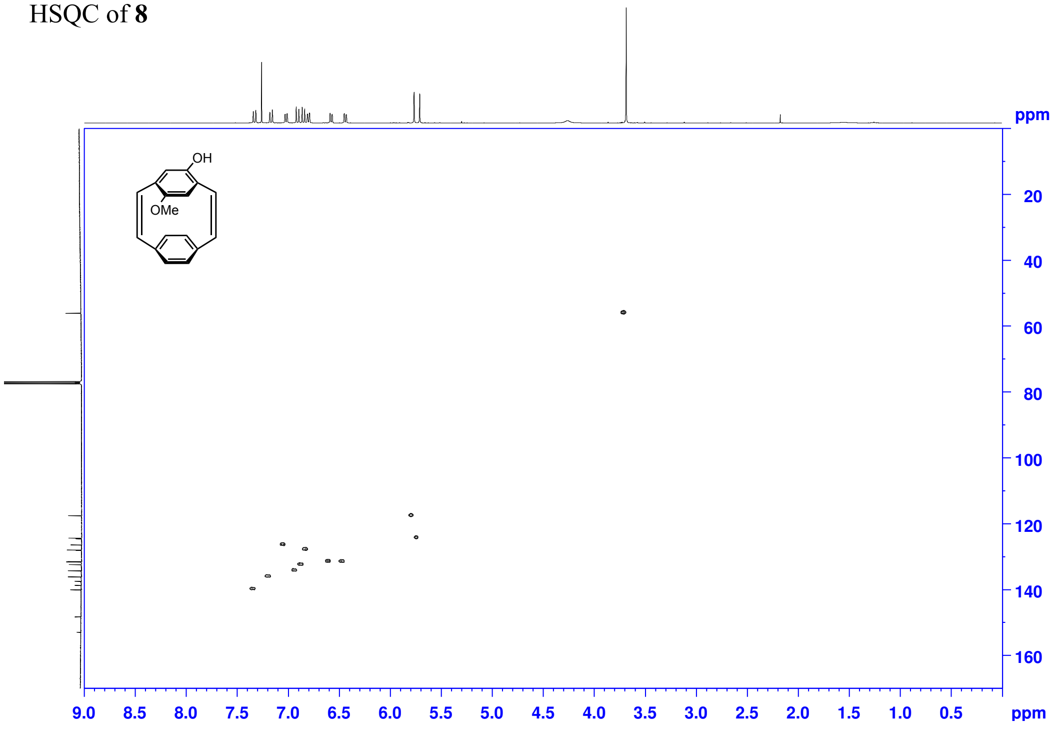

HMBC of **8**

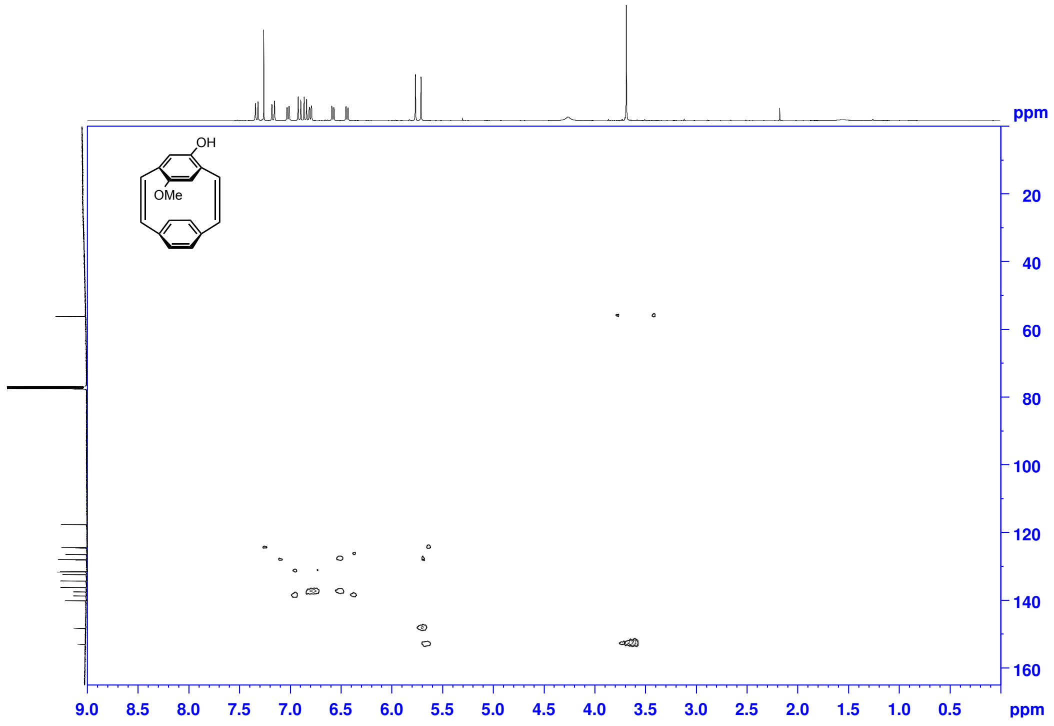

^{1}H NMR spectrum of **9**

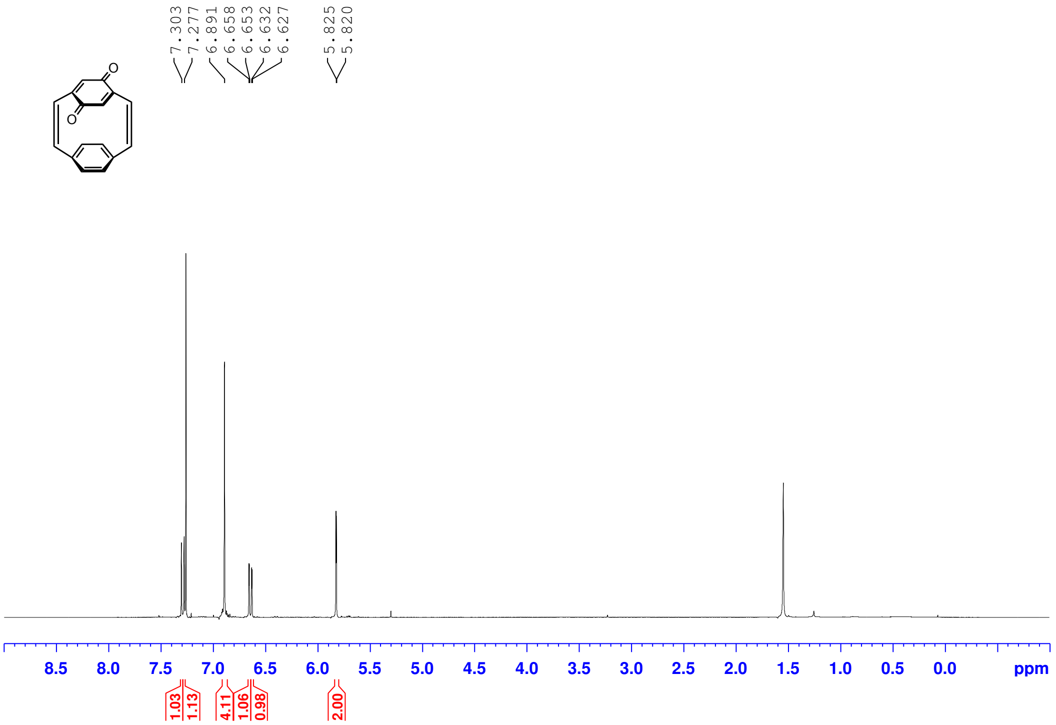

[13C] NMR spectrum of **9**

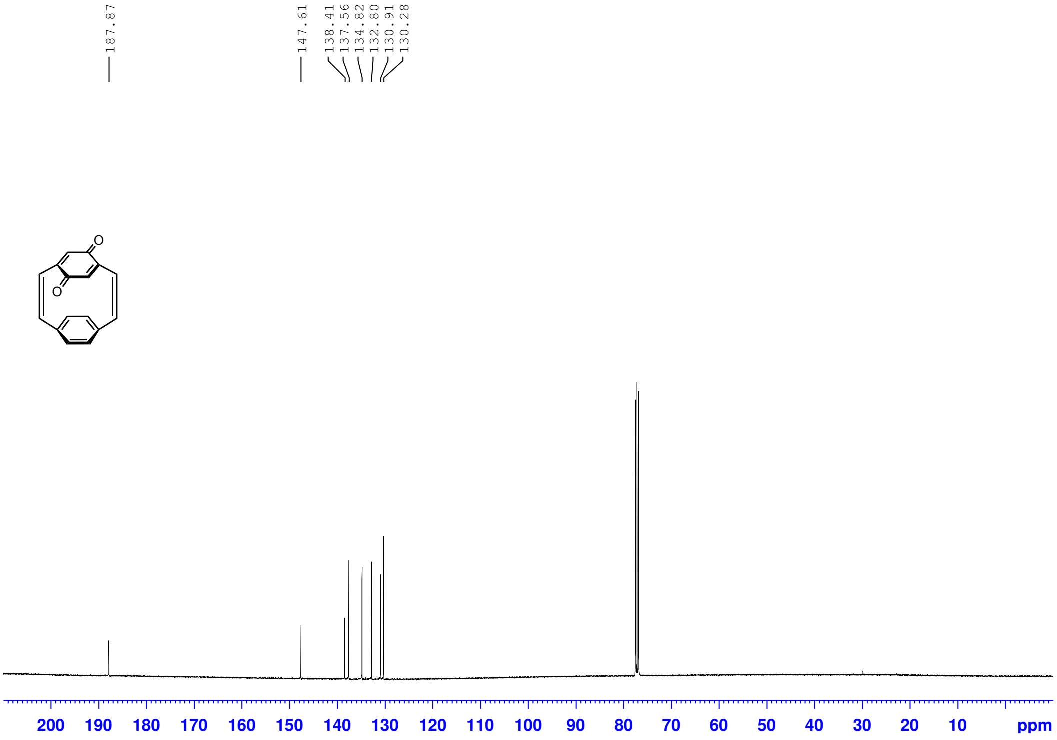

HSQC of **9**

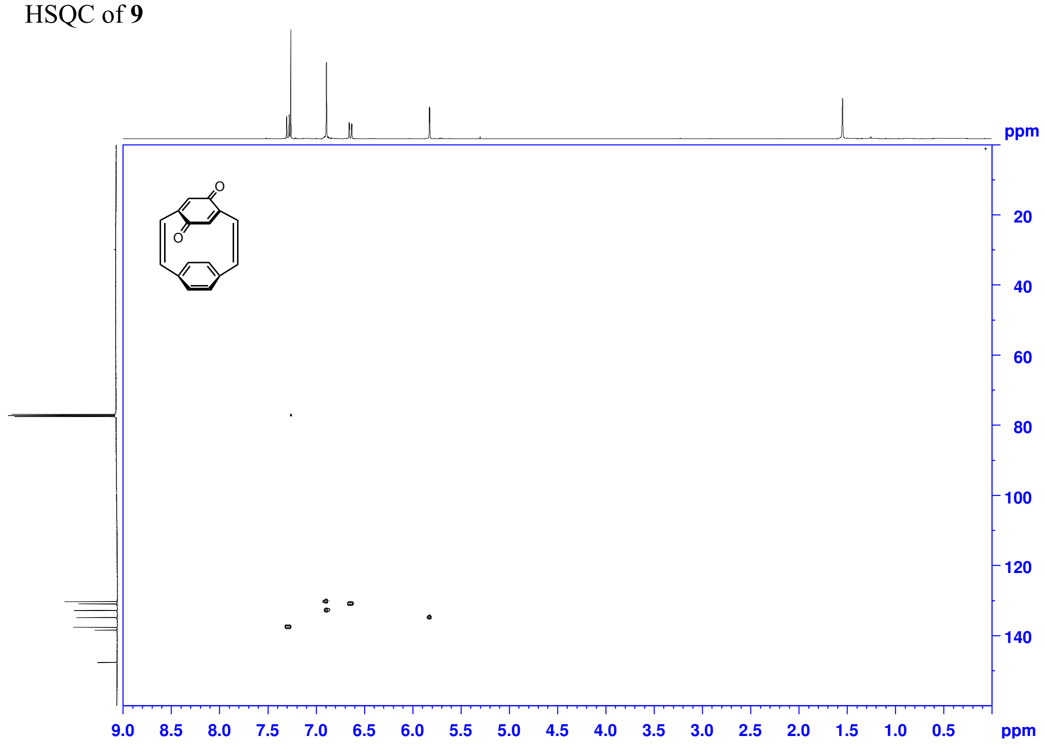

HMBC of **9**

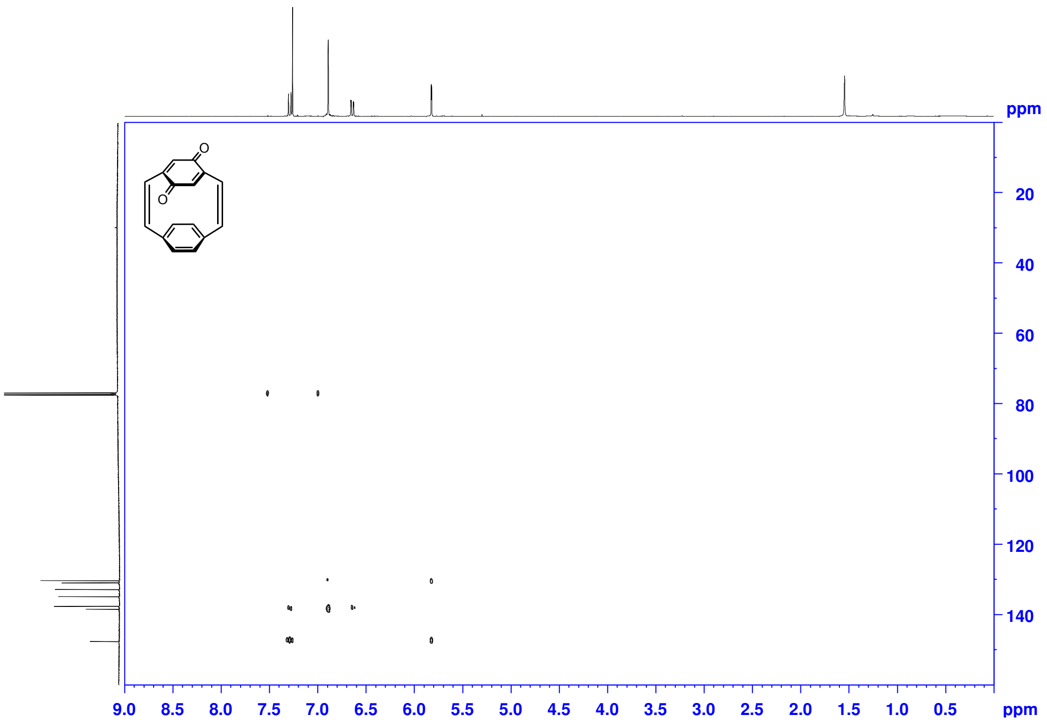

^{1}H NMR spectrum of **M1**

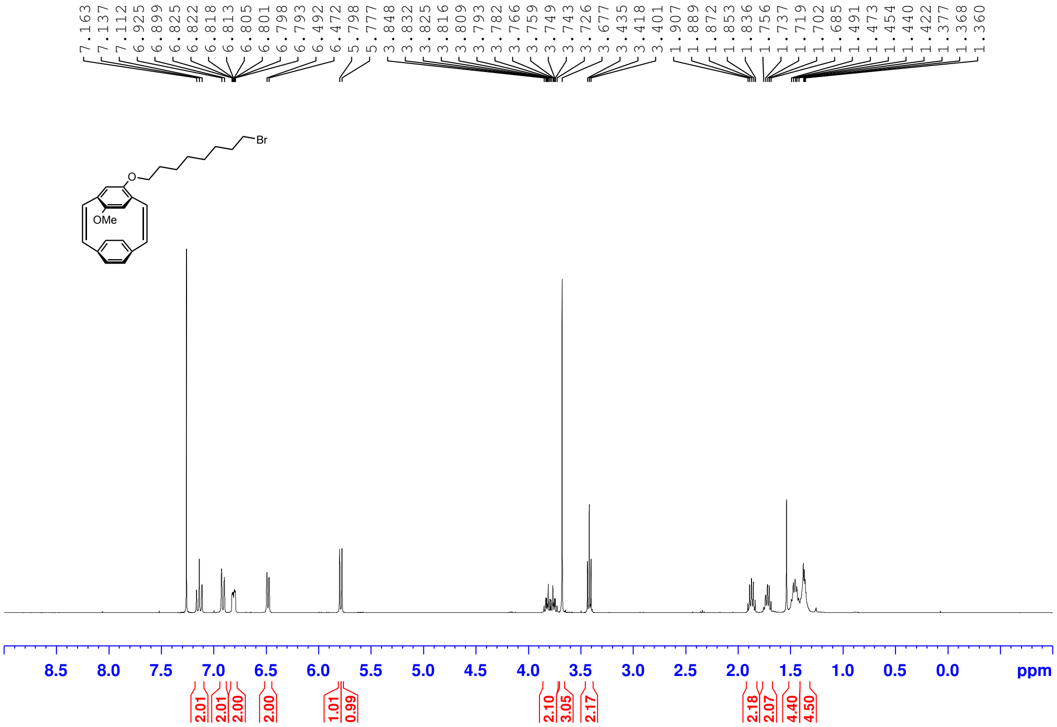

^{13}C NMR spectrum of **M1**

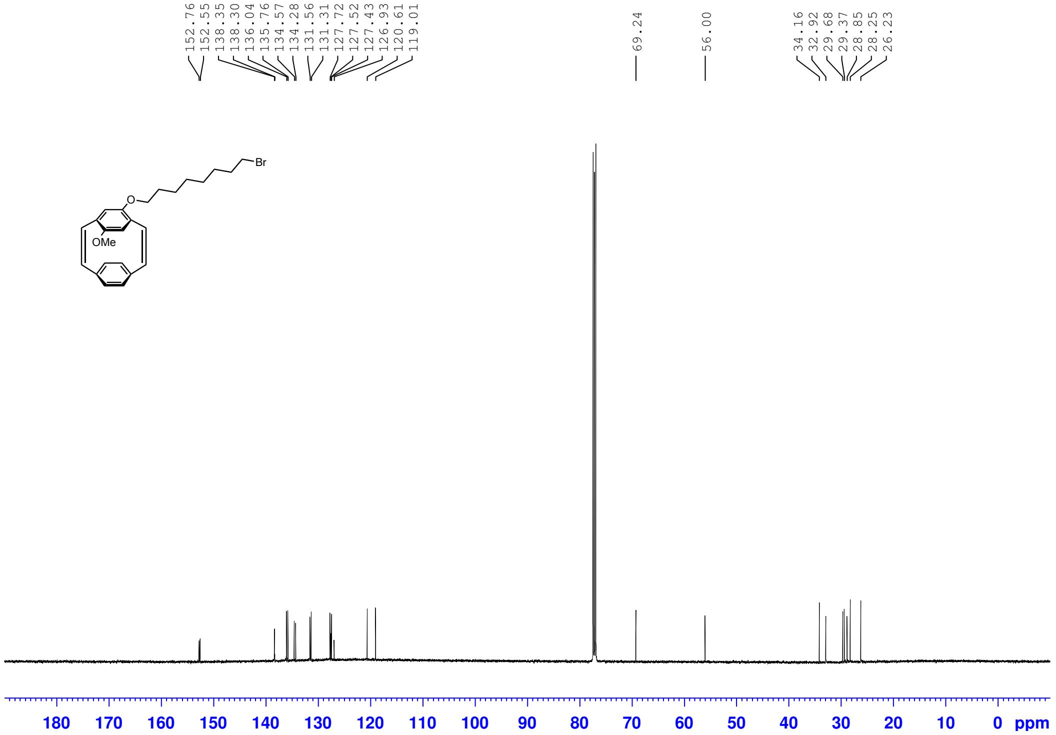

^{1}H NMR spectrum of **M2**

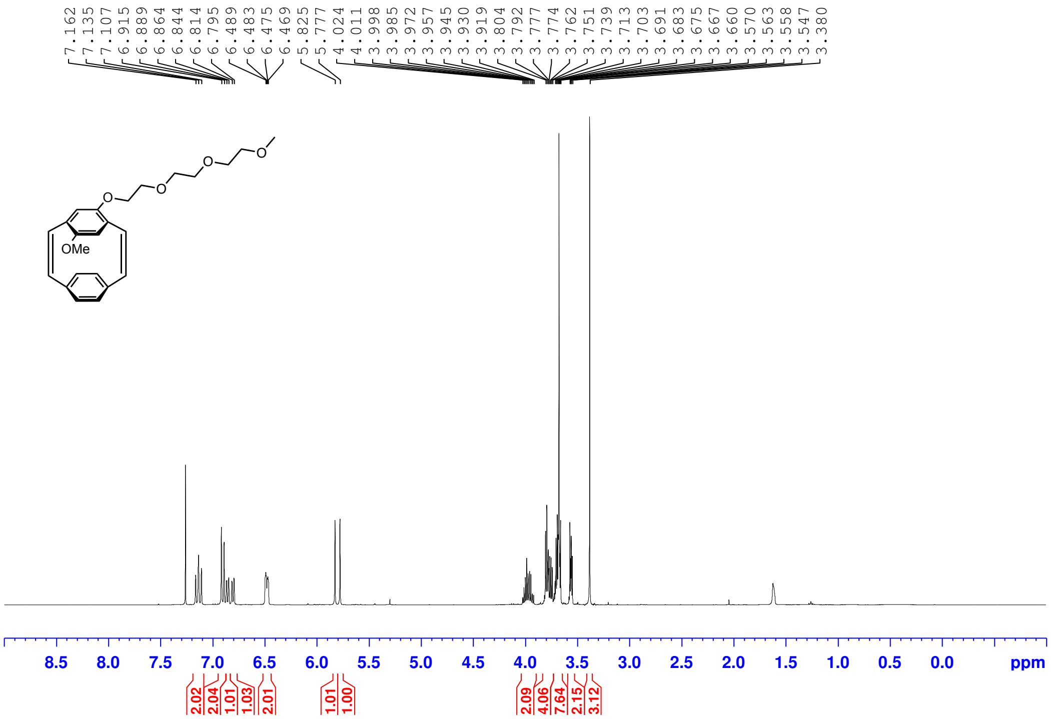

^{13}C NMR spectrum of **M2**

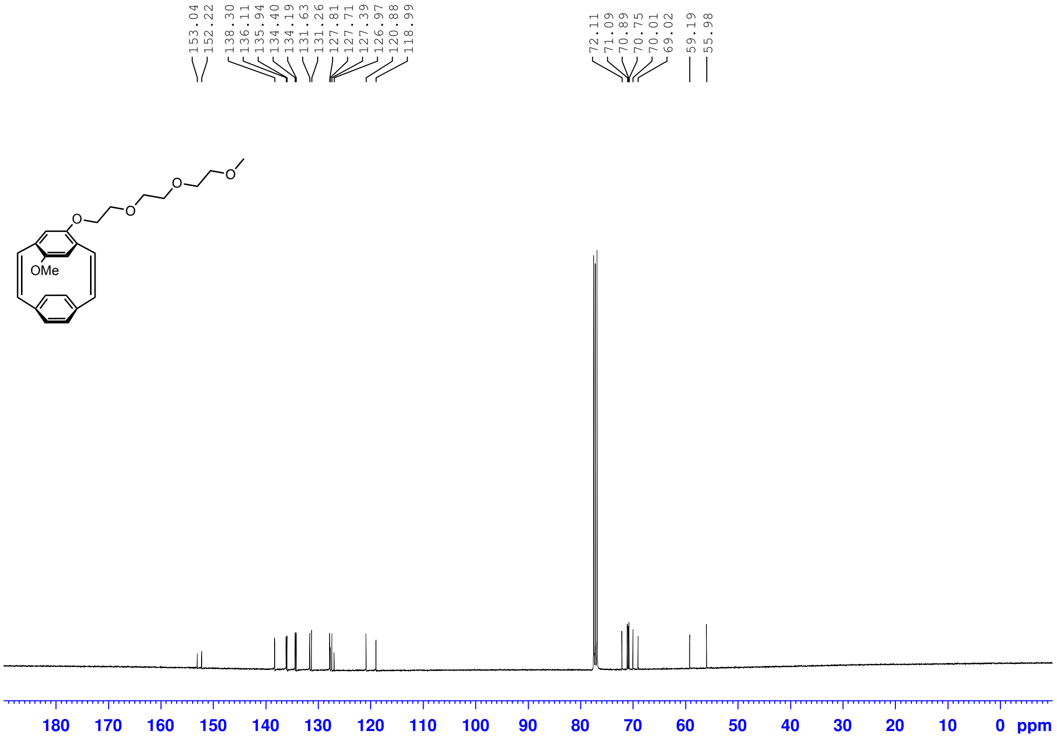

^{1}H NMR spectrum of Br-Oct-Ala-Boc

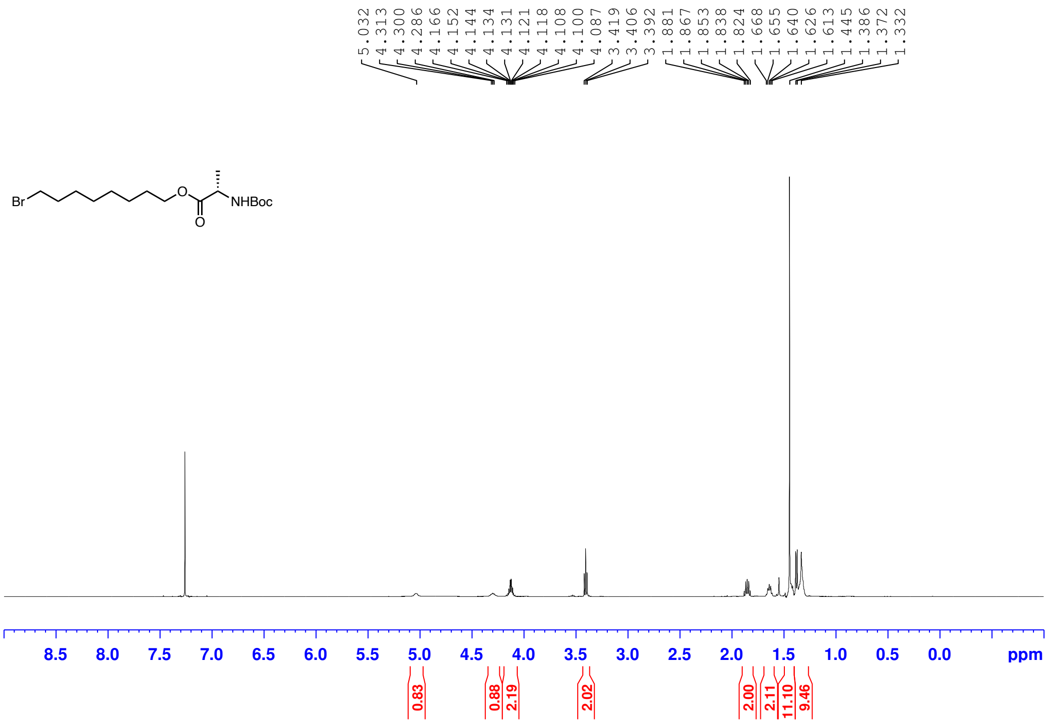

^{13}C NMR spectrum of Br-Oct-Ala-Boc

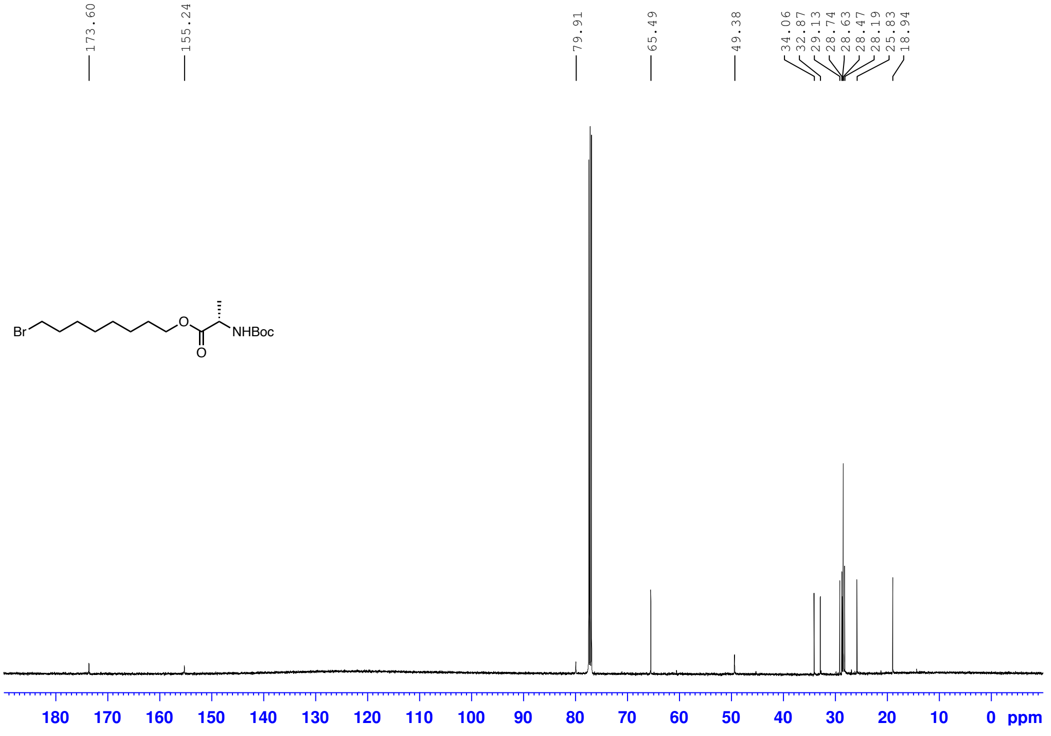

^{1}H NMR spectrum of **M3**

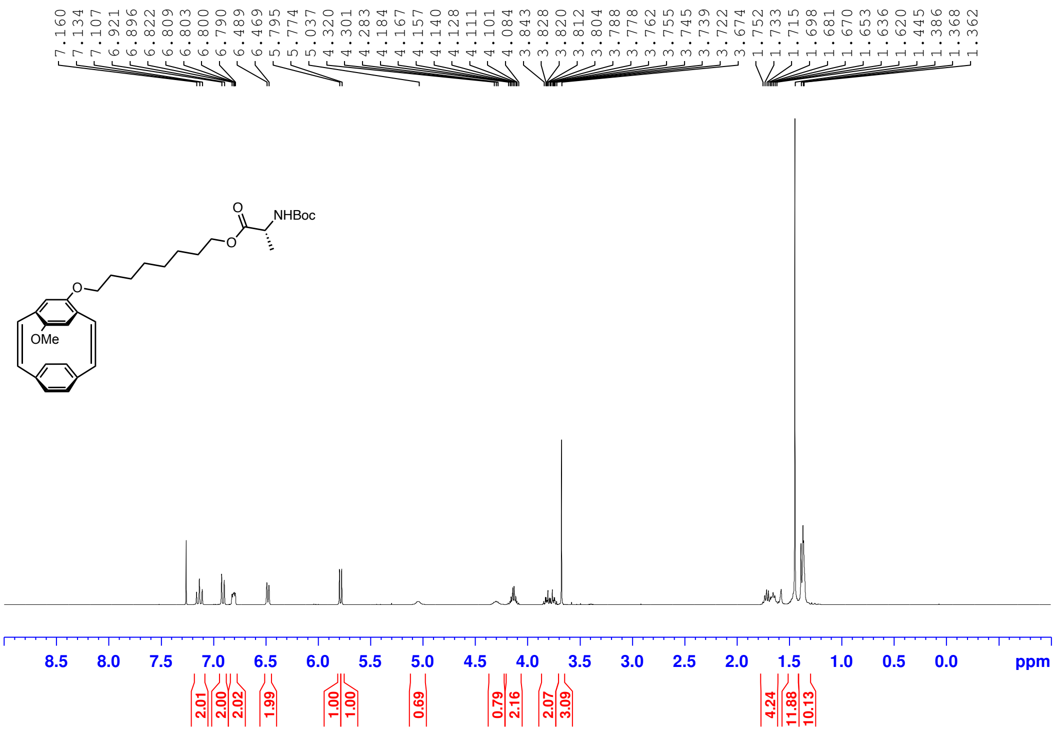

^{13}C NMR spectrum of **M3**

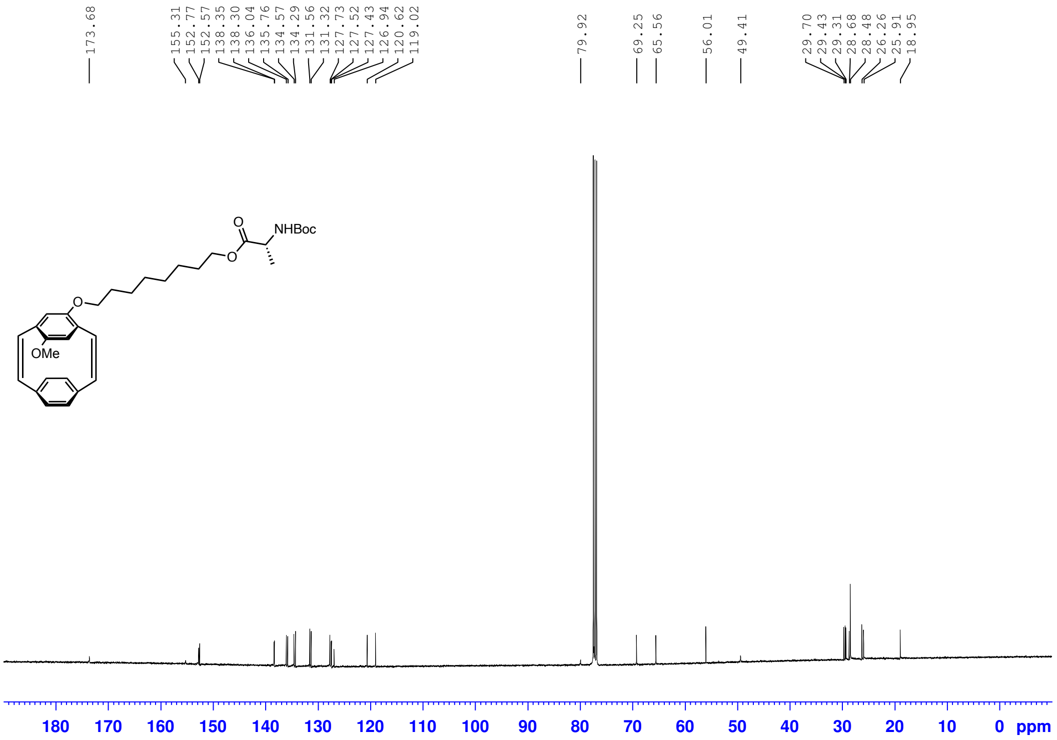

^{1}H NMR spectrum of **P1a**

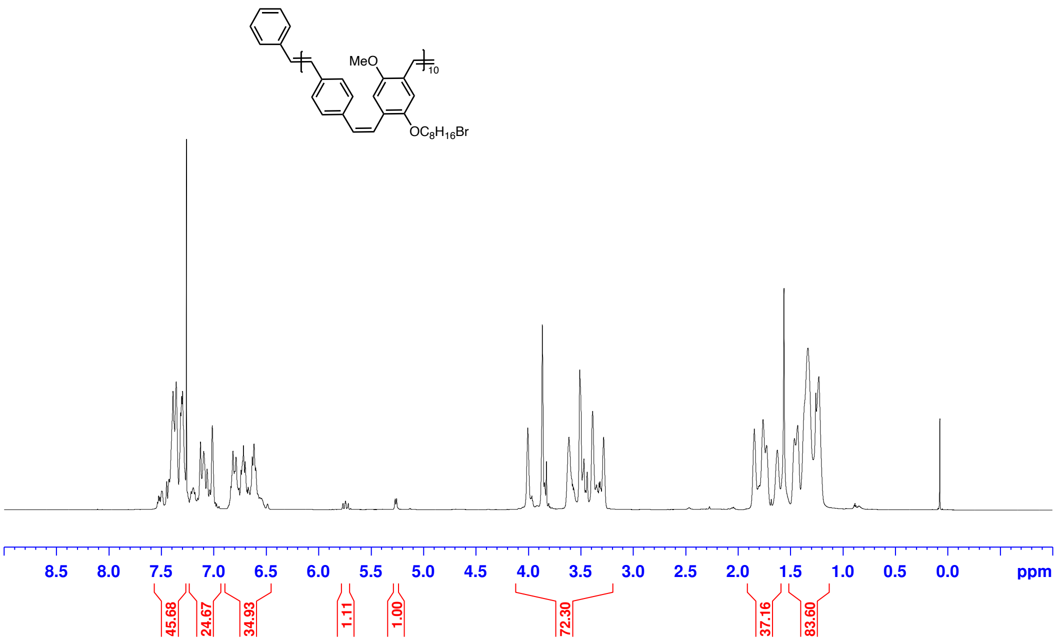

^{1}H NMR spectrum of **P1b**

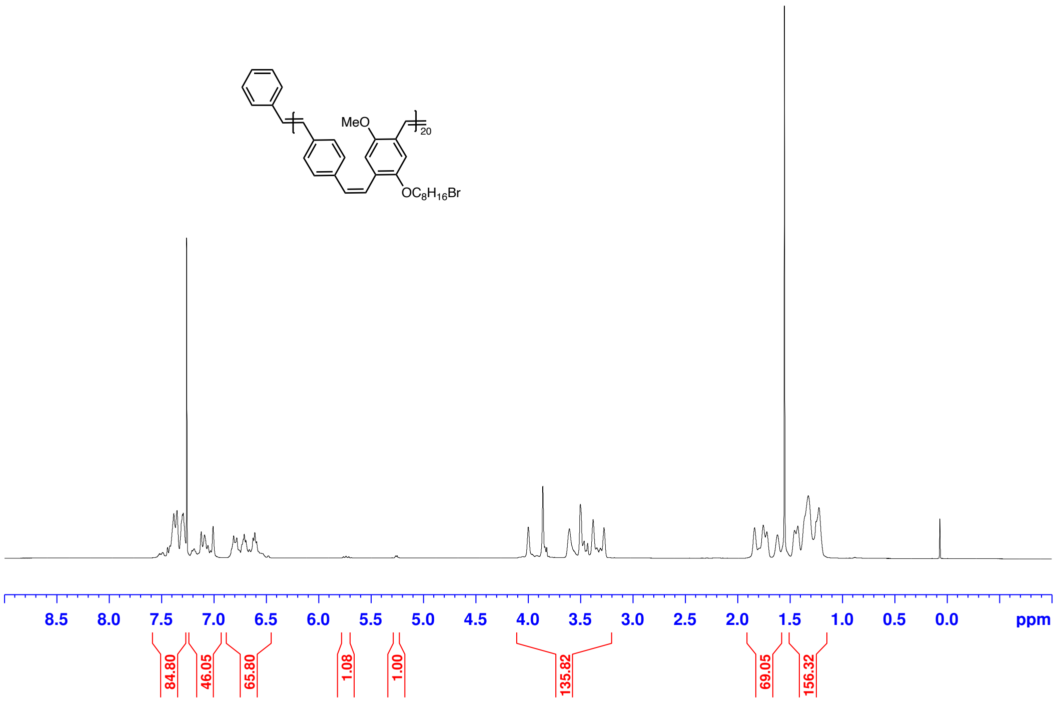

^{1}H NMR spectrum of **P1c**

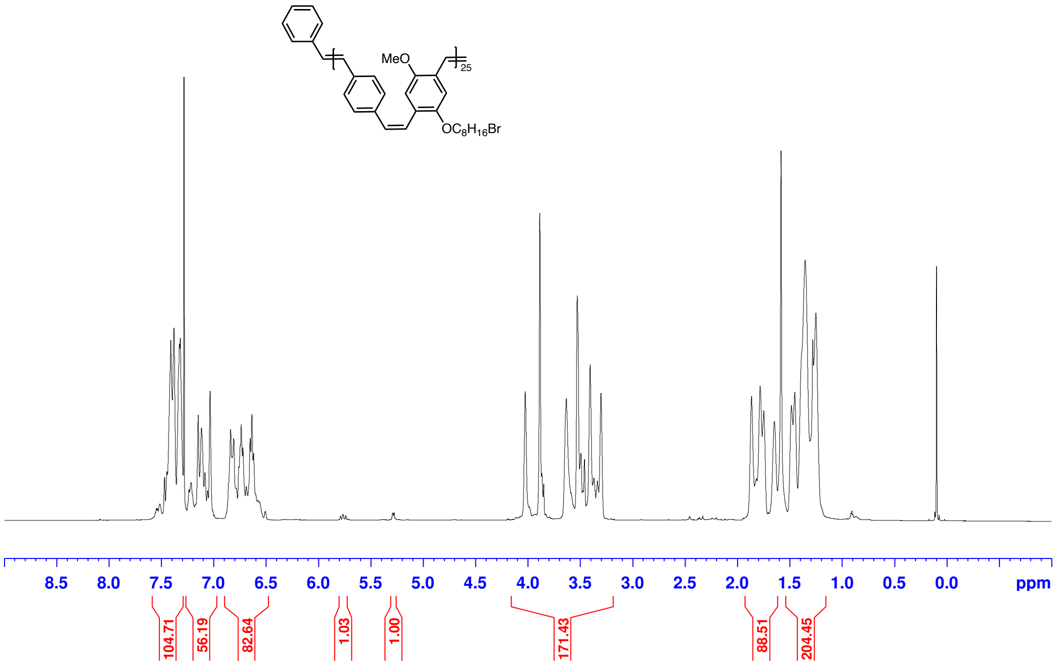

^{1}H NMR spectrum of **P1d**

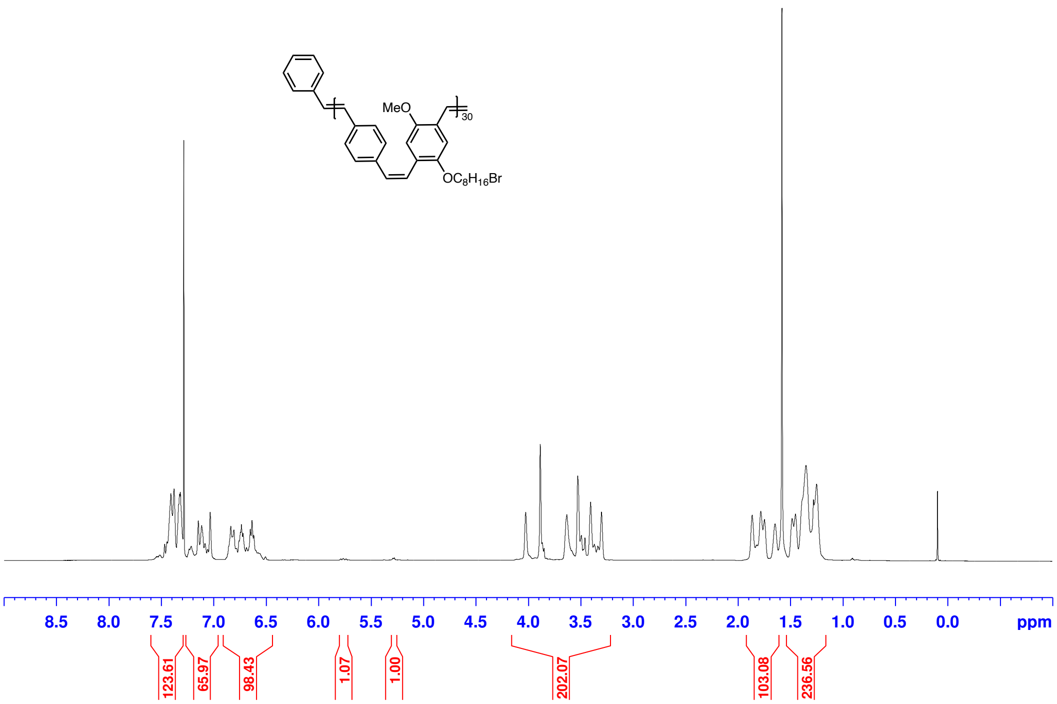

^{1}H NMR spectrum of ***trans*-P1d**

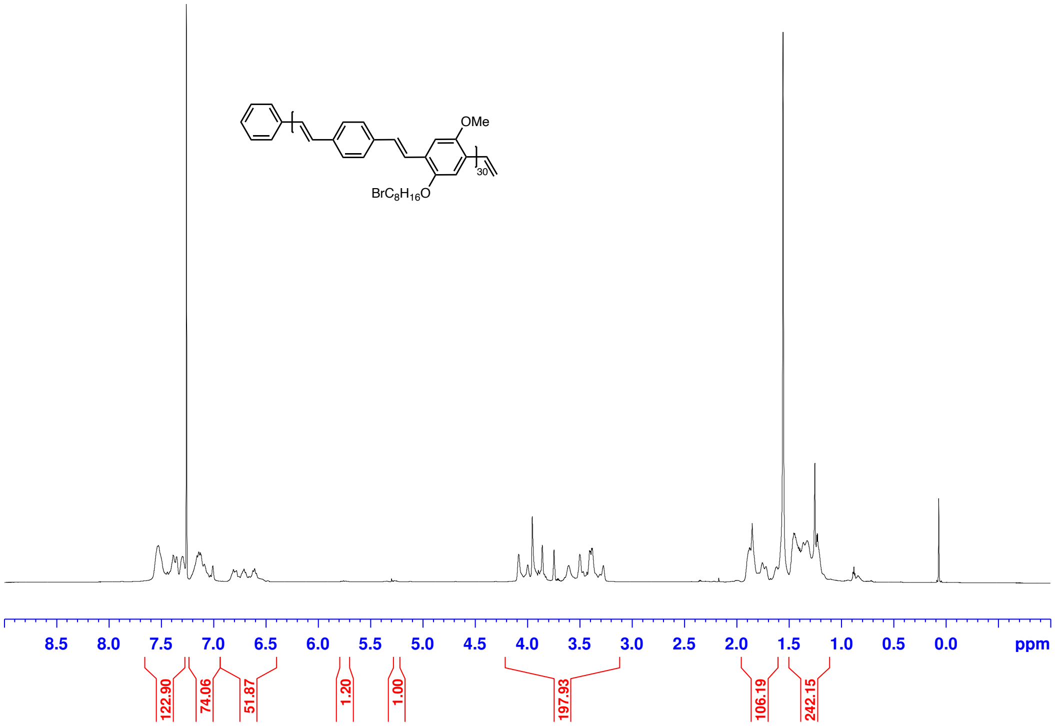

^{1}H NMR spectrum of **P1a-azide**

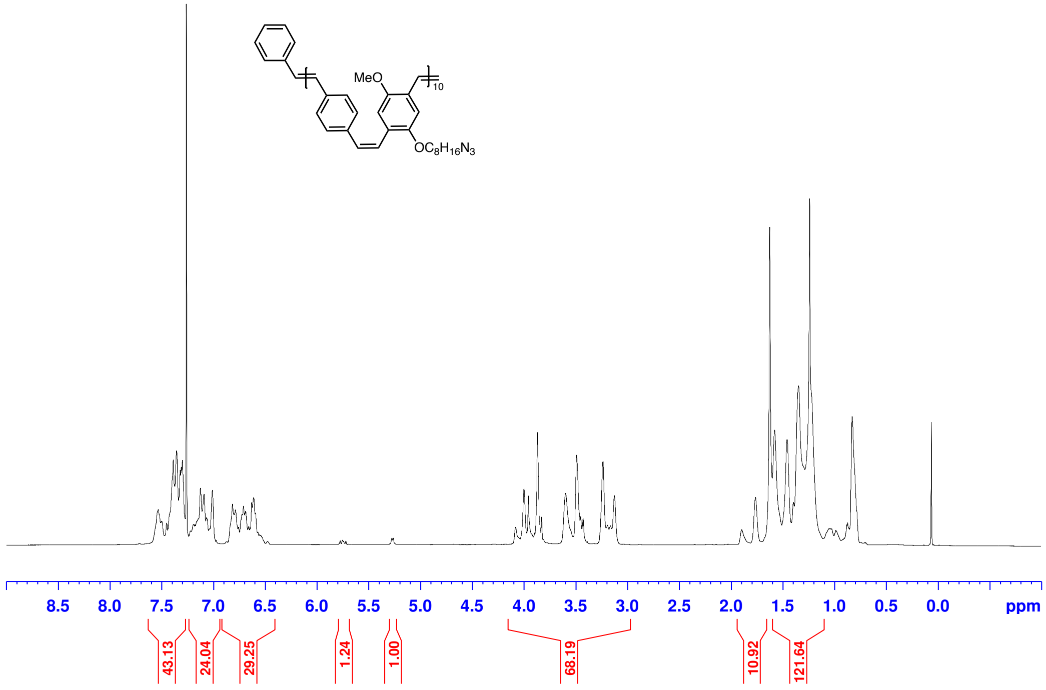

^{1}H NMR spectrum of **P1a-click**

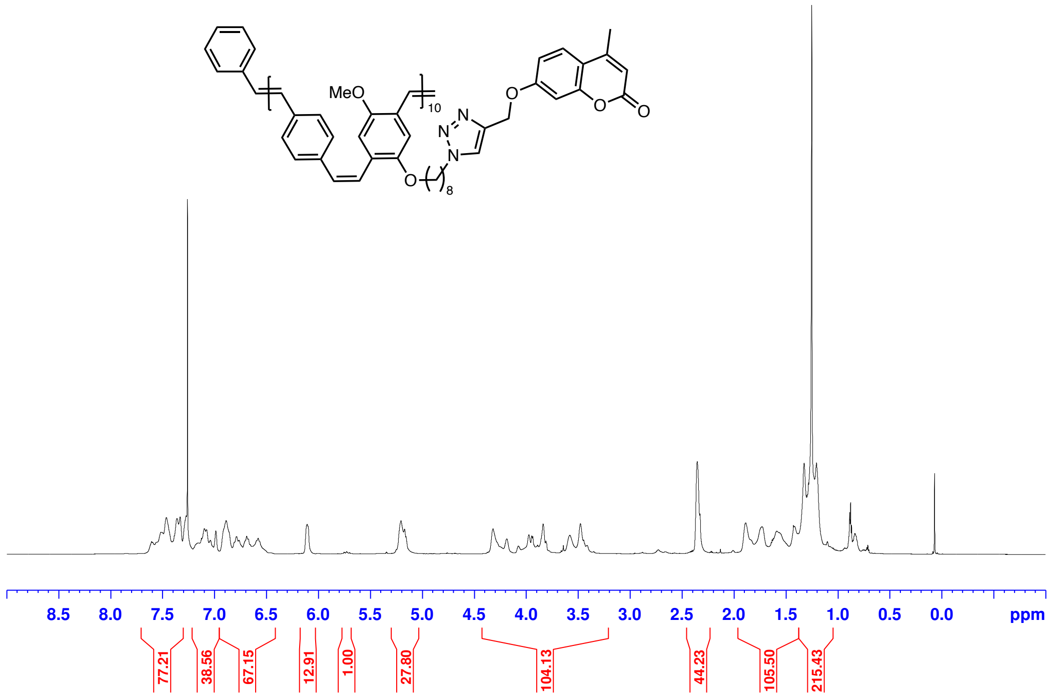

^{1}H NMR spectrum of **P2a**

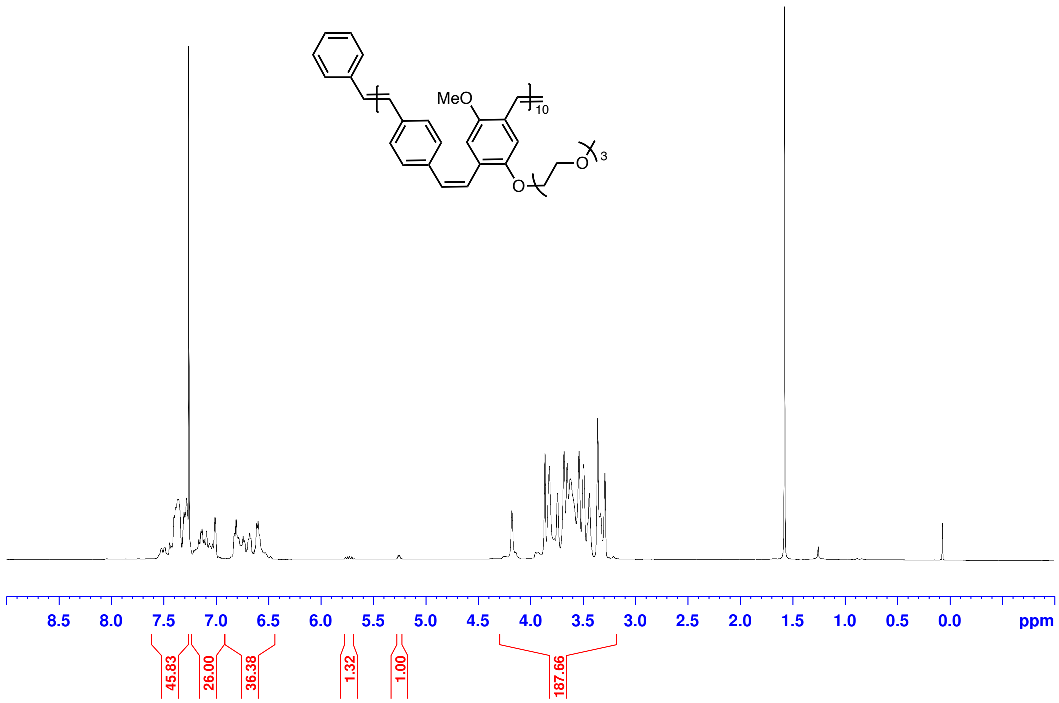

^{1}H NMR spectrum of **P2b**

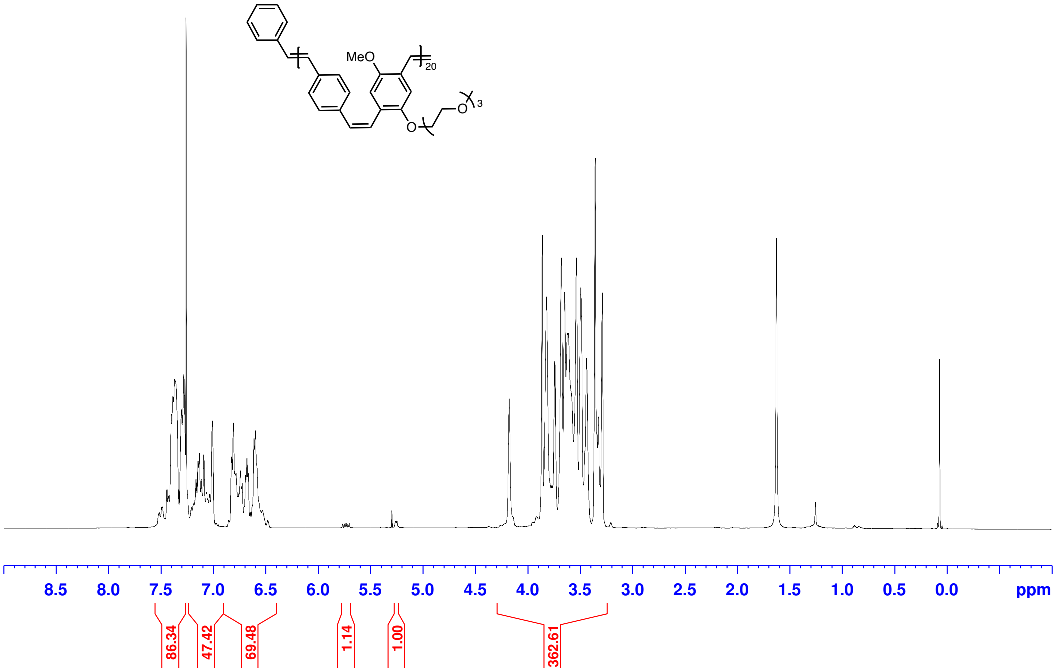

^{1}H NMR spectrum of **P2c**

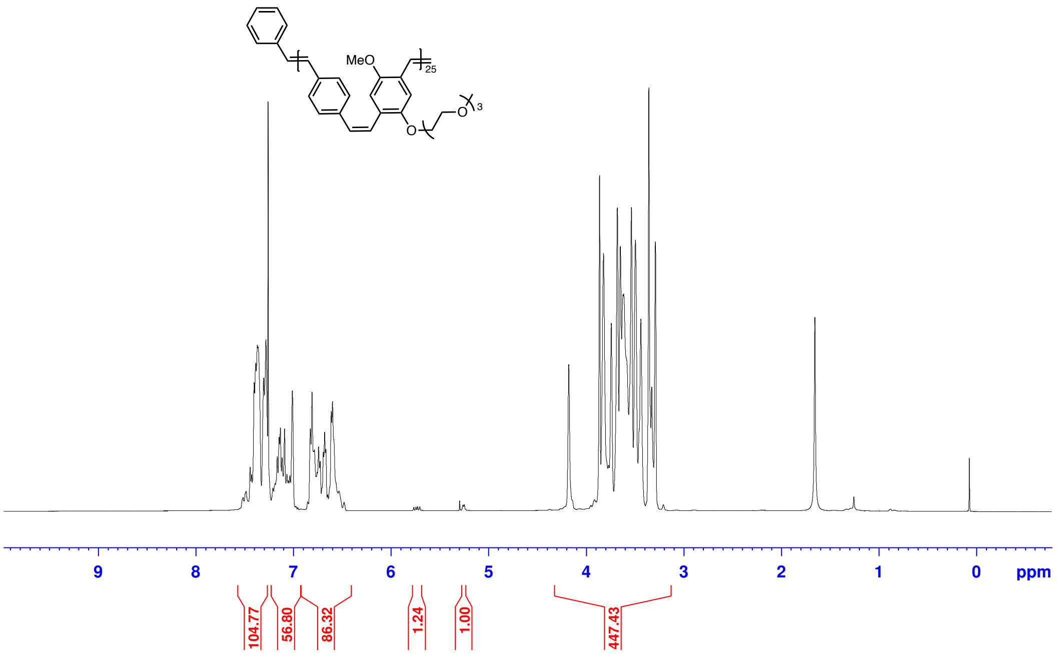

^{1}H NMR spectrum of **P2d**

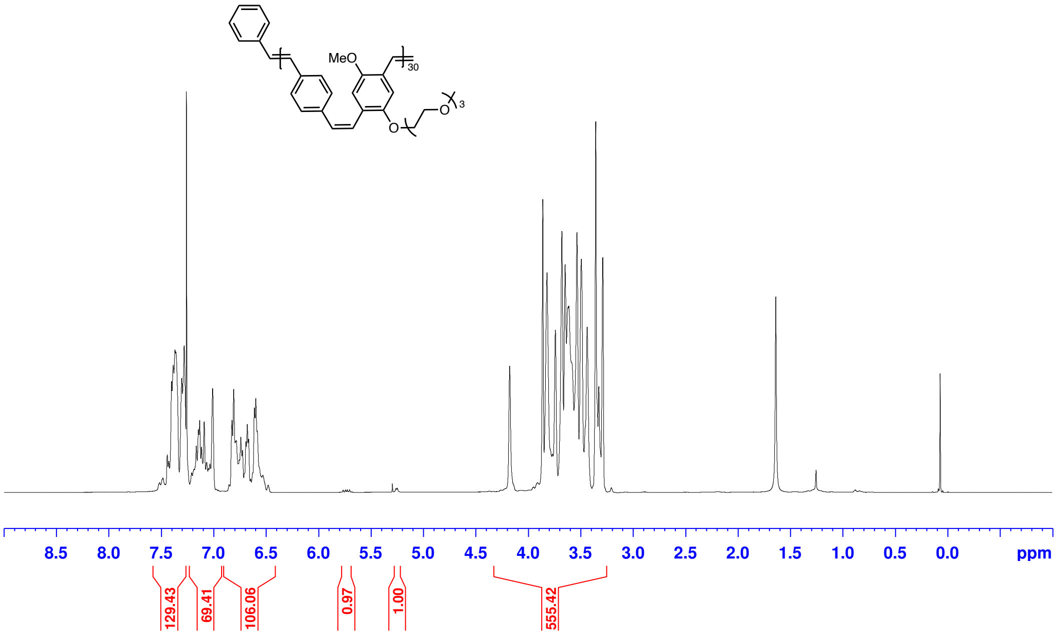

^{1}H NMR spectrum of ***trans*-P2d**

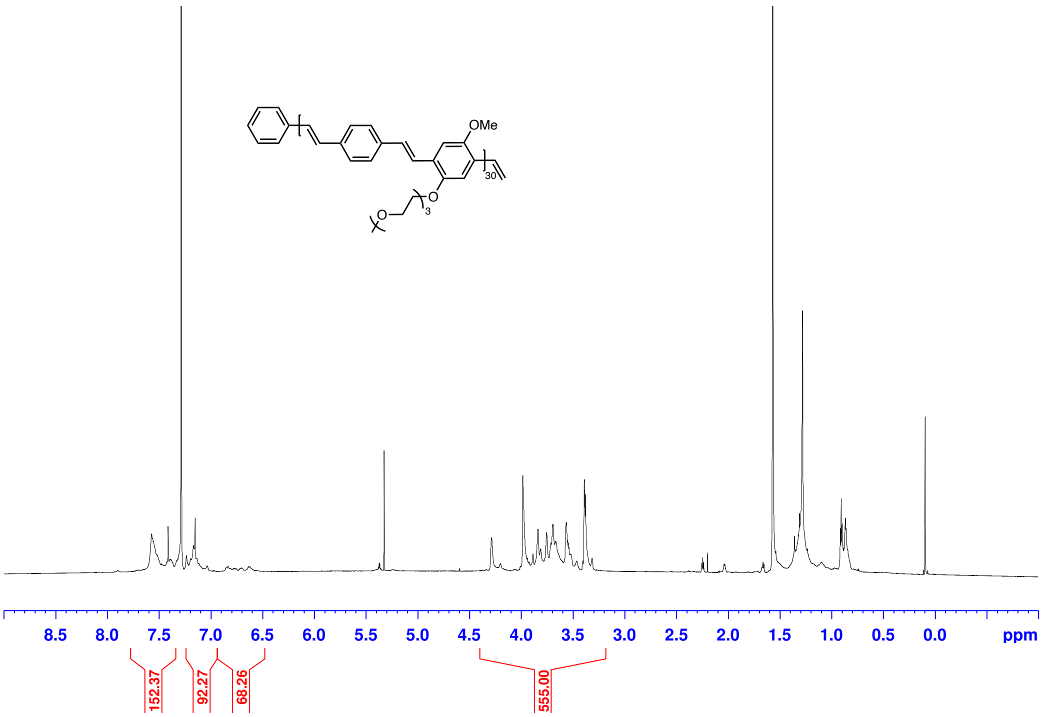

^{1}H NMR spectrum of **P3a**

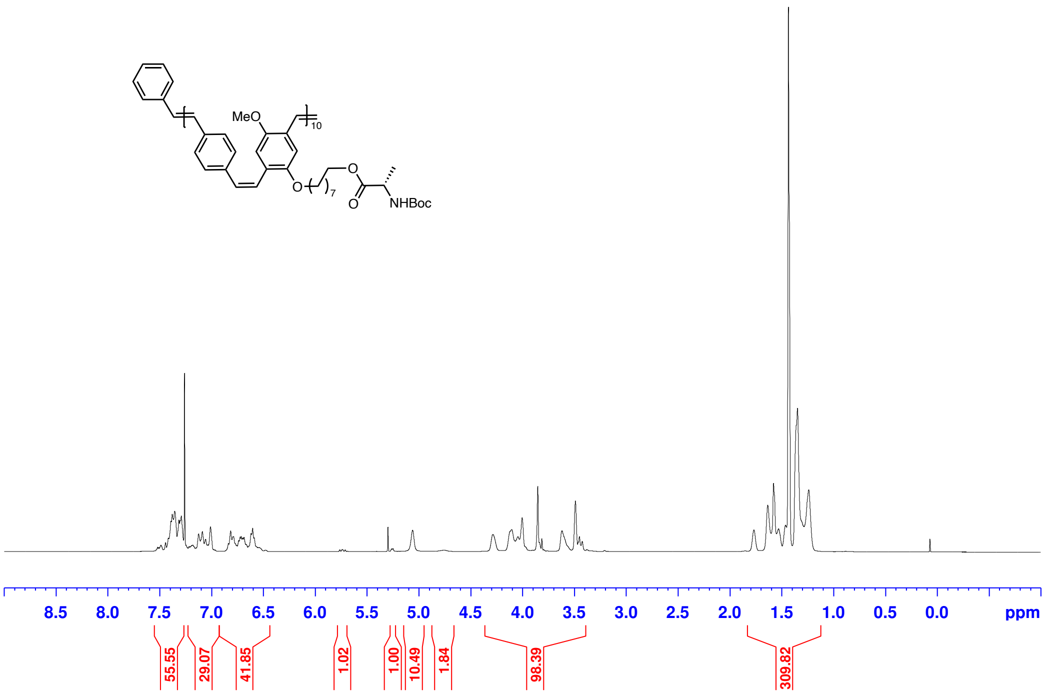

^{1}H NMR spectrum of **P3b**

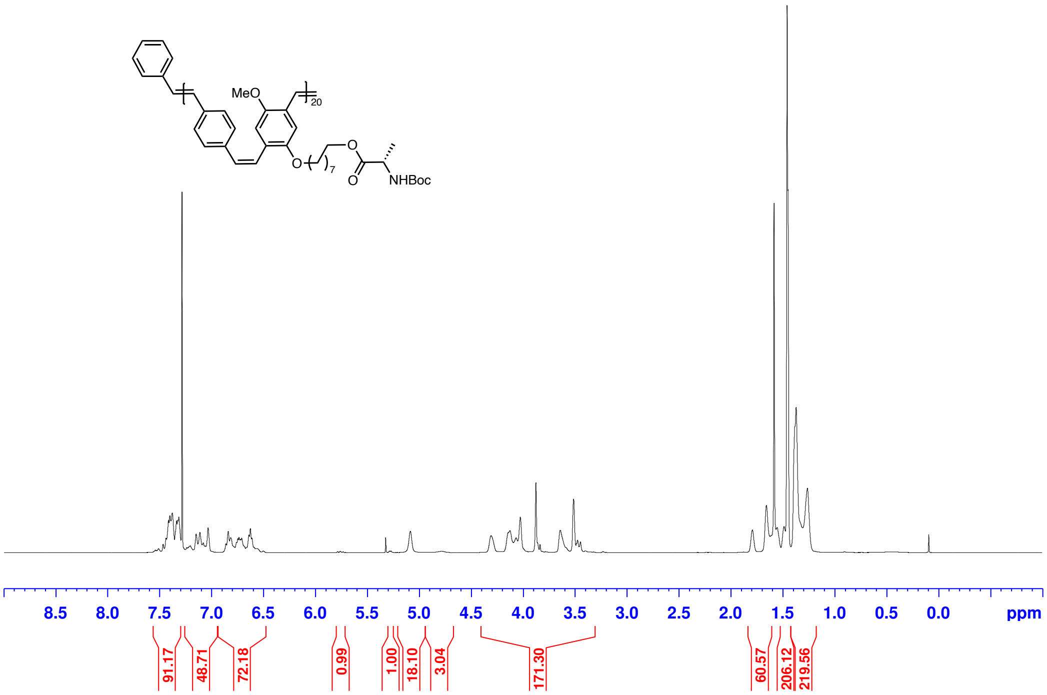

^{1}H NMR spectrum of **P3c**

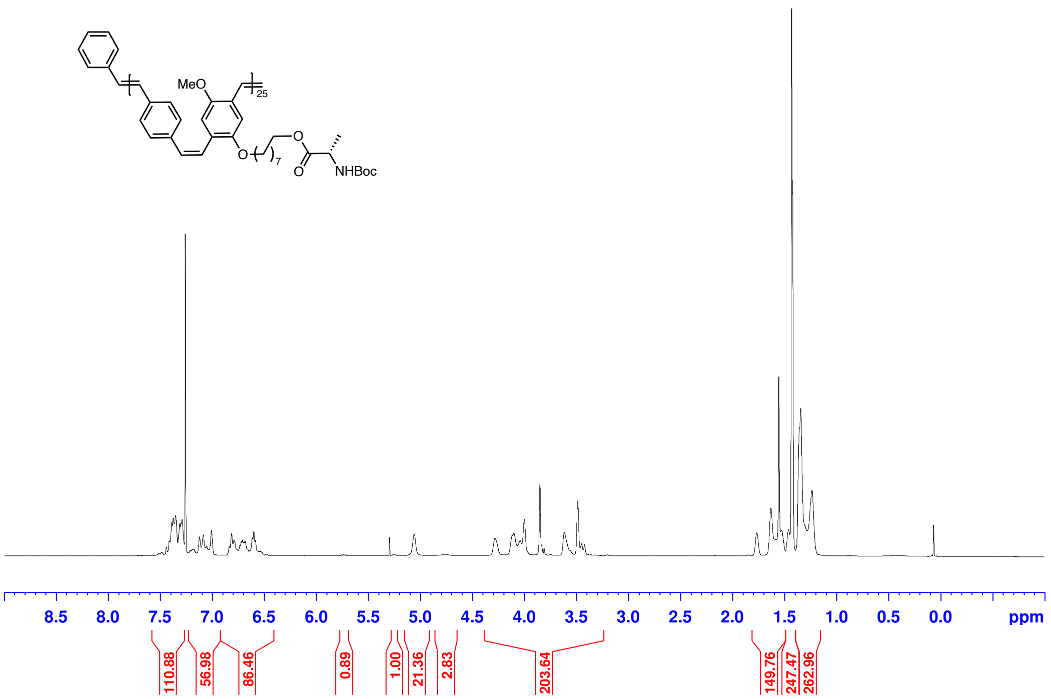

^{1}H NMR spectrum of **P3d**

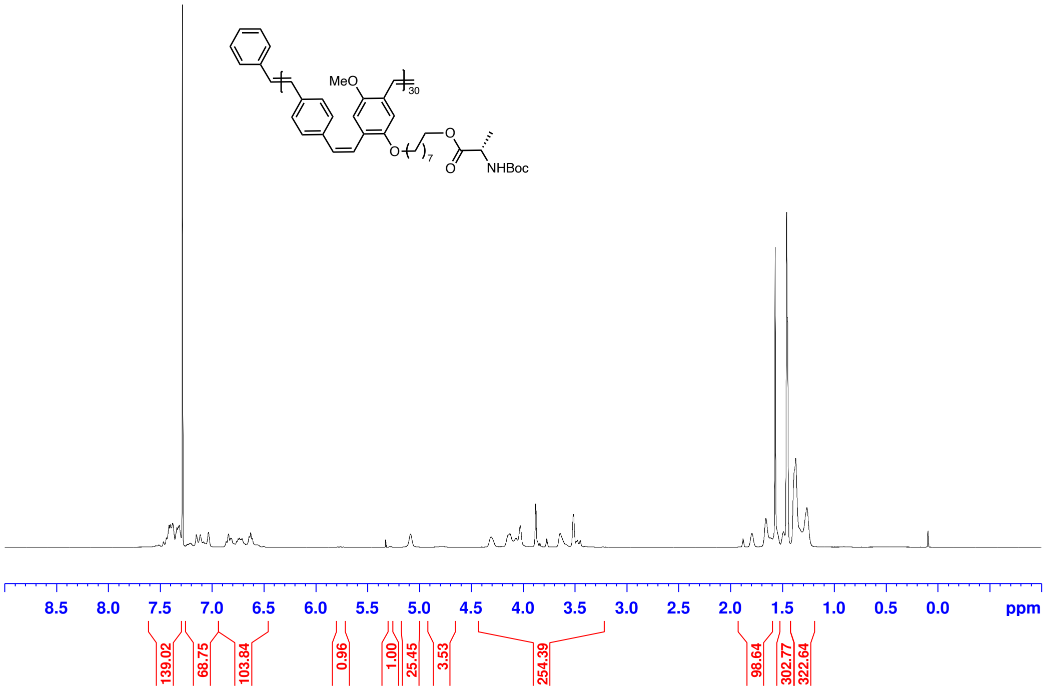

^{1}H NMR spectrum of ***trans*-P3d**

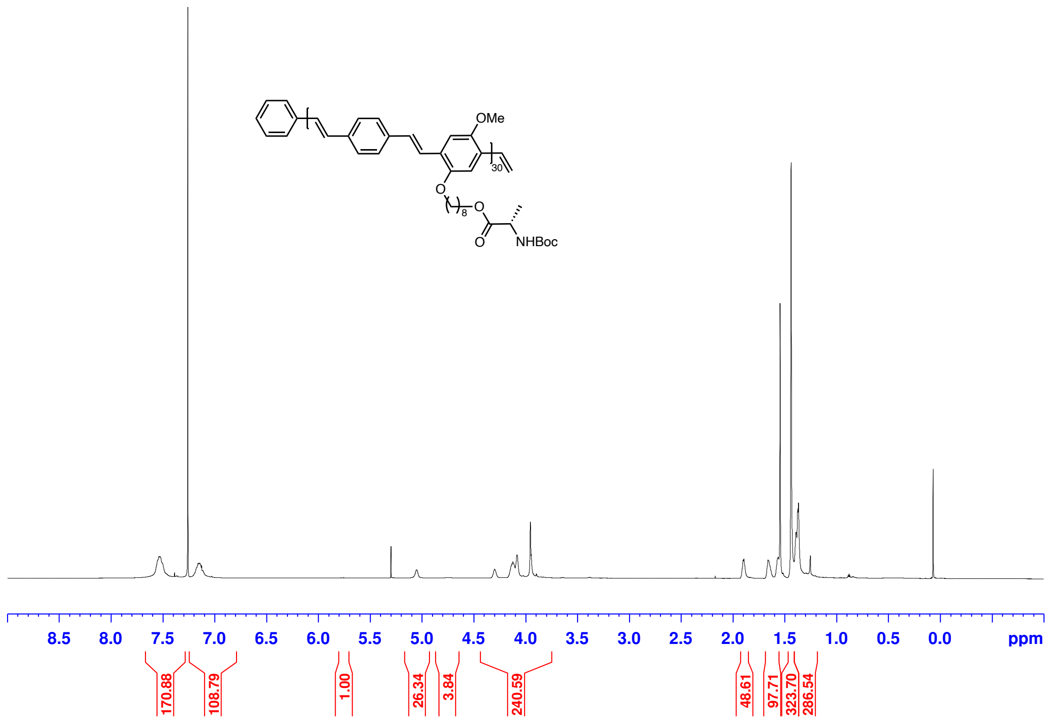

^{1}H NMR spectrum of **P4**

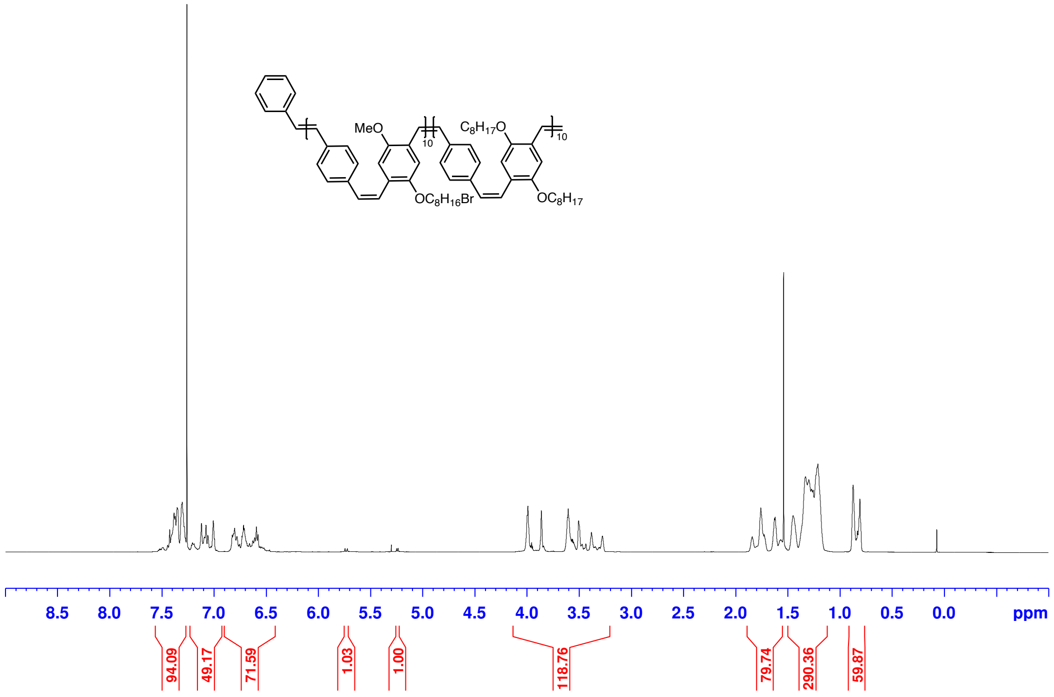

^{1}H NMR spectrum of **P5**

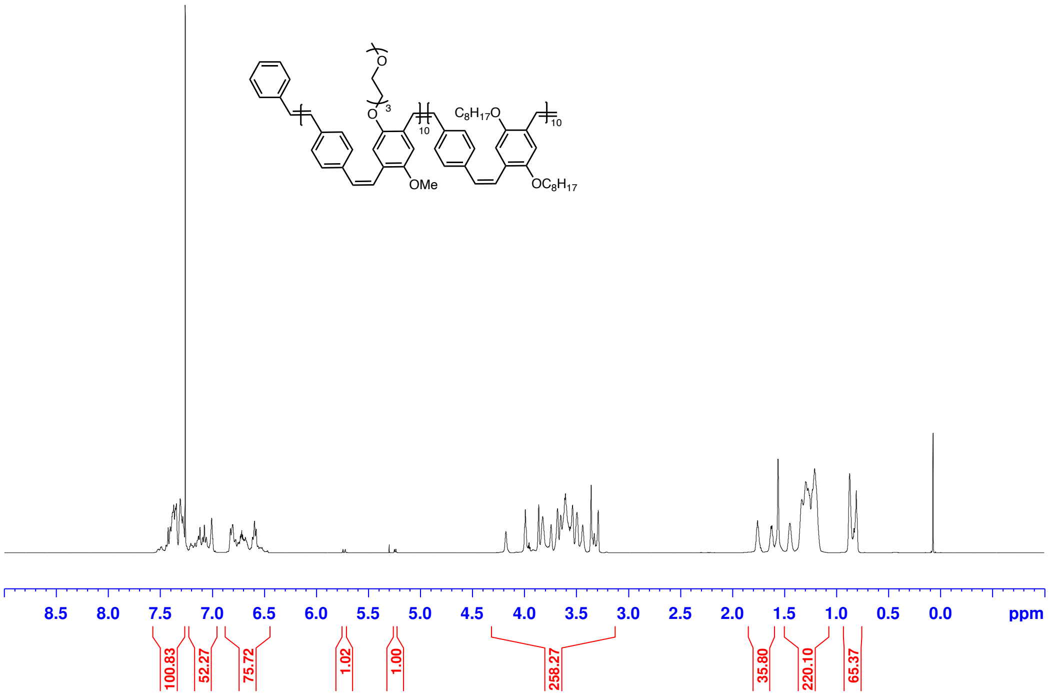

^{1}H NMR spectrum of **P6**

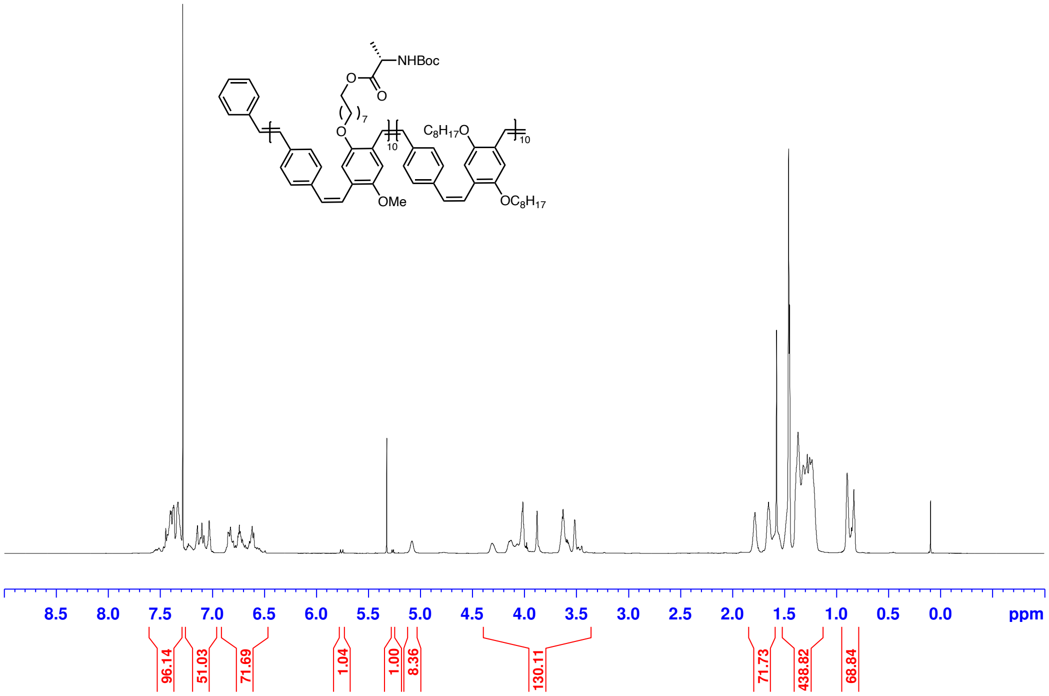

Supplement: Supplementary file 1 — mz3c00714_si_001.pdf [file mz3c00714_si_001.pdf]
